# FAVABEAN and FALAPhyl: Open-source pipelines for scalable 16s rRNA microbiome data processing and visualization

**DOI:** 10.1371/journal.pone.0331145

**Published:** 2026-04-07

**Authors:** Afnan Faridoun, Ramon Carvalho, Jacob Smith, Andrew Gibb, Lavanya Jain, Annie Zhang, Ajay Sran, Johanna Redmond, Mohammad Zubbair Malik, Monica Gibson, Anum Haider, Umar Rekhi, Anjali Bhagirath, Leigha D. Rock, Khaled Altabtbaei

**Affiliations:** 1 Department of General Dental Practice, College of Dentistry, Kuwait University, Kuwait; 2 School of dentistry, Faculty of Medicine and Dentistry, University of Alberta, Alberta; 3 Faculty of Dentistry, Dalhousie University, Halifax, Canada; 4 Department of Translational Research, Dasman Diabetes Institute, Dasman, Kuwait city, Kuwait; 5 High School Youth Researcher Summer (HYRS) Program, University of Alberta, Alberta; 6 Department of Periodontology, School of Dentistry, Indianapolis, Indiana, United States of America; 7 Department of Pathology, Faculty of Medicine, Dalhousie University, Halifax, Canada; 8 Department of Anatomical Pathology, QEII Hospital, Nova Scotia Health, Halifax, Canada; Sultan Qaboos University College of Science, OMAN

## Abstract

Reproducible and scalable analysis of 16S rRNA amplicon sequencing data remains a persistent challenge in microbiome research due to the complexity of available tools, incompatibilities between platforms, and the need for extensive bioinformatics expertise. We developed two containerized workflows—FAVABEAN (Fast Amplicon Variant Annotation, Binning, Error-correction And ANalysis) and FALAPhyl (Forays into Automating Laborious Analyses of Phylogeny)—to address these challenges. FAVABEAN and FALAPhyl are Snakemake-based pipelines designed for flexible execution across local, cluster, and cloud environments. FAVABEAN automates preprocessing, ASV inference, and taxonomic assignment using DADA2 and FIGARO, integration of taxonomic knowledge when samples are sequenced with multiple primers using SMURF. FALAPhyl supports downstream analysis including alpha/beta diversity, network analysis, and differential abundance testing, with integrated provenance tracking. We validated both pipelines using three case studies involving oral microbiome datasets. In Case Study 1, we compared oral microbiota across family members and niches, showing primer-dependent variability in ASV-based similarity and minimal reseeding from familial sources after prophylaxis. Case Study 2 analyzed dental aerosol samples, revealing no significant microbial differences between pre-, intra-, and post-procedure air. Case Study 3, a randomized trial of a nitrate mouthrinse, demonstrated no significant microbiome shifts, highlighting oral microbial stability. FALAPhyl’s integration of DAtest enabled empirical evaluation of multiple statistical tests, aiding robust differential abundance inference. FAVABEAN and FALAPhyl offer a reproducible, automated solution for 16S rRNA amplicon data analysis. Their modular design, containerization, and provenance tracking enhance accessibility and scientific rigor in microbiome research.

## Introduction

The past decade has seen an explosion in the availability and complexity of bioinformatics tools designed to analyze microbiome sequencing data. These tools have transformed how researchers interrogate microbial communities. However, with this growth has come increased fragmentation—different tools are optimized for different data types, require incompatible file formats, or demand a deep understanding of each tool’s assumptions and parameters. This has made reproducibility and provenance tracking a persistent challenge in microbiome research [1].

One major step toward addressing these challenges was the development of QIIME2 [2], a framework that emphasized transparency and data provenance. QIIME2 standardized data formats and analysis steps, making it easier to reproduce and audit workflows. However, this modular ecosystem comes with its own limitations: data must often be exported or reformatted for use in other tools, and the user must still know which steps to connect and how to parameterize them. Additionally, certain widely-used packages —such as DADA2 [3] — offer simplified and less flexible implementations within QIIME2 compared to their stand-alone versions, potentially limiting analytical resolution or customization. Other workflow management systems include mothur [4], Galaxy [5] and nf-core/ampliseq [6] provide varying degrees of automation, scalability and reproducibility but are either difficult to customize, dependent on specific platforms or require a steep learning curves for the new users.

To address these gaps, we developed and validated two complementary, open-source pipelines- FAVABEAN (*F*ast *A*mplicon *V*ariant *A*nnotation, *B*inning, *E*rror-correction *A*nd *A*Nalysis) and FALAPhyl (*F*orays into *A*utomating *L*aborious *A*nalyses of *Phyl*ogeny). These pipelines are specifically tailored for reproducible, automated analysis of 16s rRNA amplicon data enabling rapid and scalable workflows with minimal intervention. The impetus of the pipeline is to enable rapid generation of results suitable for exploratory data analysis with minimal to no post-execution hands-on interventions. The two open-source pipelines are built on Snakemake [7], making them highly scalable across different computational environments.

FAVABEAN focuses on preprocessing, denoising and taxonomic assignments of amplicon data with integrated functionality for datasets sequenced using multiple primer sets. FALAPhyl, is designed for downstream analysis and visualization, including alpha/beta diversity, network analysis, and differential abundance testing, while maintaining full data provenance via containerization and workflow tracking. Both pipelines are implemented using Snakemake, a scalable workflow management system and utilize several open-source packages, accessible under different platforms such as R, Python, and the shell [8–18]. Importantly, they are containerized via Docker, ensuring version control and computational reproducibility across platforms-from local machines to cloud computing environments.

In the following sections, we demonstrate the functionality and capabilities of these pipelines using three diverse case studies, based on original and secondary analyses of publicly available datasets. We highlight how both FAVABEAN and FALAPhyl streamline complex analyses, reduce manual effort, and improve reproducibility — making them valuable tools for microbiome researchers across disciplines. The code for both pipelines are available on GitHub (github.com/khalidtab/) and are available on Docker Hub as precompiled images (hub.docker.com/repositories/khalidtab). Relative to the aforementioned popular pipelines, our two pipelines provide complementary and distinct features which are discussed below.

## Materials and methods

**Fig 1:**
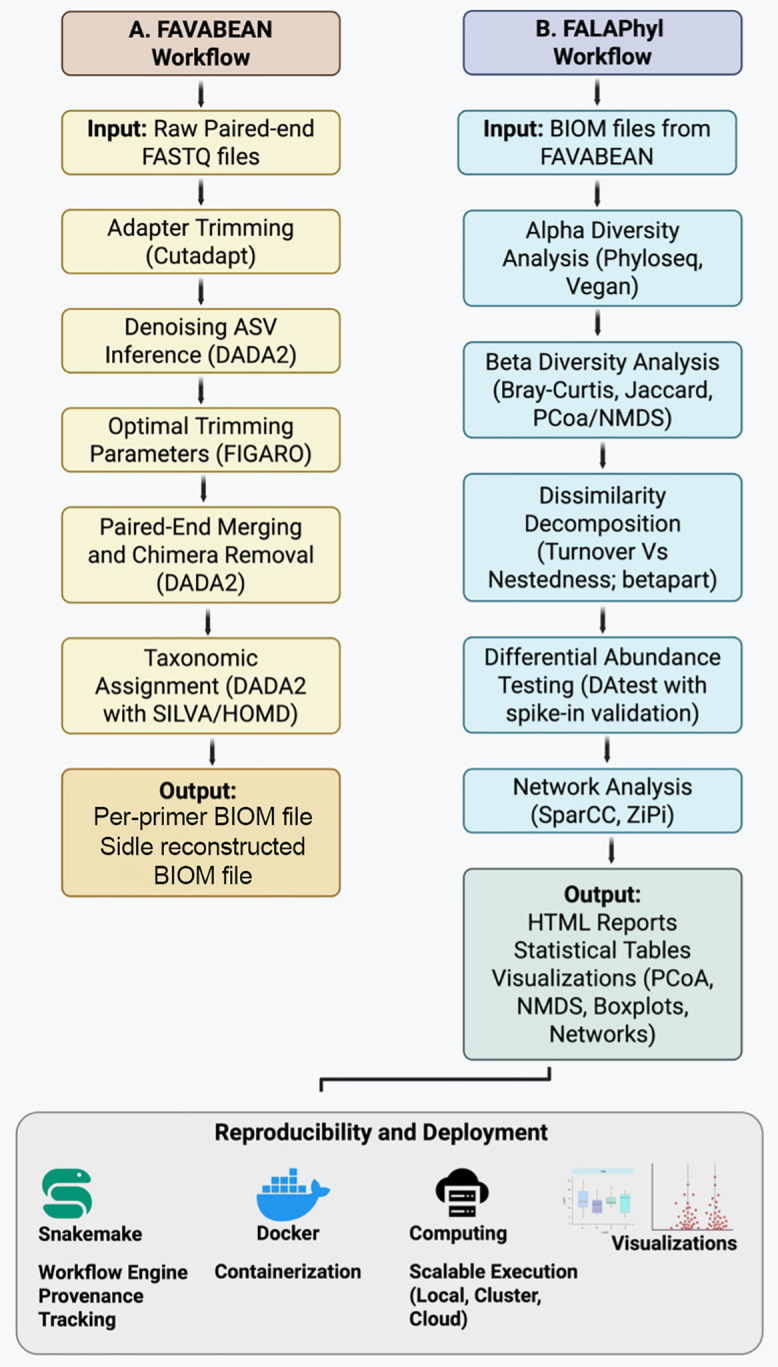
Overview of the FAVABEAN and FALAPhyl workflow for 16S rRNA amplicon analysis.

Detailed explanation for both pipelines is available in supplementary. FAVABEAN and FALAPhyl are implemented using Snakemake [7] as its foundation, with various connecting scripts written in R, Python, and shell (Fig 1). Snakemake itself allows the pipelines to be implemented in various scales from a single computer to a cluster, to a cloud implementation without needing to change the workflow. Although the precompiled pipelines are available as a Docker image which are pinned to specific versions of the libraries that are Linux compatible, re-compiling the pipelines dependencies to other operating systems are possible through removing the pinned files in the workflow/envs/ folder. The folder also contains the versions of the packages that the pipelines are designed to work with. Snakemake also examines the rules of the pipeline and the sequential dependencies of the rules on each other, then executes the rules using the computational resources provided. If enough resources are available, non-sequential portions of the analyses are automatically executed to allow for faster completion of the steps, and better utilization of the computational resources.

Within both pipelines, running the start command provides all the functions that are available to run the pipelines. Running this command also writes the requisite files for running the two pipelines; the metadata are provided as text files, and parameters are supplied to the pipelines as YAML configuration files. Description of each parameter in the YAML files are extensively explained within the files. Samples are provided in metadata text files, and parameters and metadata are specified in the pipeline the FAVABEAN pipeline, taxonomic binning reference libraries are automatically downloaded if they have not been downloaded previously.

### FAVABEAN

The pipeline’s input is FASTQ files. The files are analyzed with FIGARO [19] for the best parameters to funnel to DADA2 [3] for generating Amplicon Sequence Variants (ASVs). The pipeline supports two options funnels from FIGARO; “highest coverage” which retains the greatest number of sequences in the samples at the expense of more sequencing errors analyzed by DADA2, or “lowest errors” which retains only the highest quality sequences at the expense of reducing the coverage within the samples. Each amplicon of a specific 16s rDNA hypervariable region preferentially targets areas which can distinguish some bacteria with high accuracy compared to others. Therefore, to get good coverage of taxa present in the bacteriome, one is expected to sequence the same sample under multiple primer sets targeting different hypervariable regions. However, this inflates the presence of certain taxa if they are detected under multiple primer regions. This pipeline handles this situation automatically. If two or more primers are used to sequence the same samples for larger coverage of the taxonomy [20], then the pipeline automatically utilizes a Short Multiple Reads Framework (SMURF) to reconstruct the sequences using the taxonomy rank of the sequences into lower taxonomic ranks, after constructing a Maximum Likelihood model of their probabilities on reference databases [21]. The implementation within this pipeline utilizes their Python code refactoring that was packaged in a QIIME2 plugin (Sidle – [22]). The implementation within FAVABEAN strips the QIIME2 dependencies, while keeping the code and logic of the implementation largely intact. This allows them to be automatically generated, with the non-sequential steps be run in parallel after Snakemake’s rules dependency evaluation. These BIOM files can then be used in the second pipeline to generate preliminary analyses.

### FALAPhyl

The entry points for the pipeline is through requesting the following general analyses: alpha-diversity, beta-diversity, differential abundance, network topography, and breakdown of Bray-Curtis and Jaccard dissimilarities into its two components [18]. Moreover, the pipelines provide within-sample versions of alpha-diversity, differential abundances (DA), and breakdown of the two dissimilarity matrices. By utilizing Snakemake’s reporting capabilities, provenance is maintained by packaging the input files, the results, and the environments by which the results are generated into a file that can be shared to other researchers or attached to manuscripts.

The results will demonstrate the usage of these pipelines with three diverse case studies to show how the pipelines can be utilized.

## Results

### Case study 1: Microbial similarity in family members

**Fig 2:**
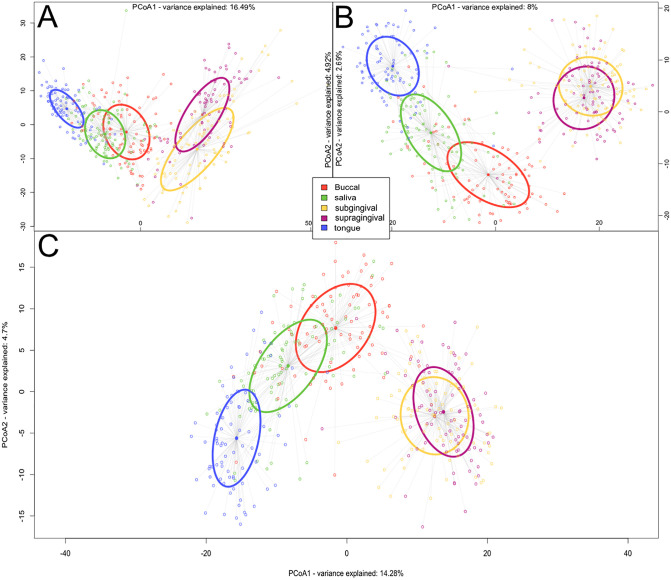
Beta diversity (PhILR distances) Principal Coordinate Analysis (PCoA) plots. **A)** ASVs of V1-V3, **B)** ASVs of V4-V5, and **C)** Sidle-reconstructed taxonomies. Graphs were adjusted for clarity by modifying axis labels inside the graphs and legend placement.

To illustrate the functionality utility of the two pipelines, we analyzed data from families where one child received dental prophylaxis, with 1 follow up appointment. Detailed methods are provided in Appendix (S1 File). The study was approved by the University of Alberta Research Ethics Board (Project No. Pro00106647). Samples were deposited in NCBI SRA (PRJNA1159177). In summary, oral samples from parents and their children were collected across multiple oral niches to examine microbial similarity and niche-specific differences within family units. One child underwent dental prophylaxis and was re-sampled one-week post-treatment. Samples were sequenced using two primer sets, V1-V3 (27F-519R) and V4-V5 (515bF-926R), which differ in taxonomic coverage and bias towards certain bacterial species [20]. Given that differences were previously examined at a species level in the Human Microbiome Project [23], we were interested in identifying these differences at the ASV level, as well as the species taxonomy level. FAVABEAN pipeline was executed using the “pairedtaxonomy” workflow after accounting for the sample structures in a mapping file (see appendix (S1 File) for details and tutorial on how to run the pipeline). This generated three count tables in BIOM format: one for each primer, as well as a table of reconstructed taxonomy, at the depth of the taxonomic rank identifier in the database of that particular sequence, after Sidle reconstruction (taxonomy assignment through DADA2, and Sidle-based reassignment was done using eHOMD V15.22) [24]. The BIOM files were then processed through FALAPhyl for alpha and beta diversity analyses using both cross-sectional and paired-sample modes. Beta diversity analysis (PhILR distance) revealed distinct clustering of supragingival and subgingival plaque samples from other oral niches across all three count tables (Fig 2).

To evaluate how primer choice affects observed microbial similarities across niches, we compared ASV-level analyses by individual primers with Sidle-reconstructed taxonomy-based results. In particular, we focused on identifying which biological sample type is most similar to subgingival and supragingival plaque. Sidle-reconstructed taxonomy analysis (Table 1) showed that subgingival and supragingival plaque are equally far from buccal mucosa (FDR adjusted p-value 0.1). Buccal mucosa was the most similar to supra- and subgingival plaque, then saliva, then the tongue. However, when examining ASV-level differences by primer, the patterns diverged. With the V1-3 amplicon, saliva samples were the most similar niche to plaque, then buccal mucosa, then tongue. While tongue showed the greatest separationall, the overall distinctions were more modest than those seen in the taxonomy-based analysis. In contrast, with the V4-5 primer, the pattern was reversed: buccal samples were closest to plaque, while tongue was the most dissimilar. These findings highlight that conclusions about biological similarity can vary significantly depending on the primer used and the resolution of analysis (taxonomy vs. ASV). Based on these findings, the V4–V5 primer set was selected for further analysis.

**Table 1:** Statistics of the pairwise PhILR distances of three samples (buccal, saliva, or tongue) to the supra- and sub-gingival samples. Note that PhILR is not boundary constrained between −1 and 1, therefore distance comparisons should only be done within the same feature resolution level.

				Distances to subgingival plaque samples		Distances to supragingival plaque samples	
Samples	Feature resolution level	Z-score	FDR adjusted p-value	Median (Min-Max)	IQR	Median (Min-Max)	IQR
Buccal	Sidle	−1.23	0.1	47.45 (26.20-65.84)	5.4	47.67 (26.84-64.28)	5.37
Saliva		−6.15	4.14E-10	48.51 (28.64-73.02)	5.41	49.05 (31.67-68.19)	4.99
Tongue		−6.95	1.93E-12	50.34 (5.37-35.67)	5.375	51.12 (32.62-60.82)	4.69
Buccal	V1-3	7.03	1.23E-12	85.68 (50.33-109.23)	10.4	84.67 (49.87-109.55)	11.68
Saliva		6.312	1.59E-10	85.47 (50.68-105.42)	9.79	84.75 (52.22-106.36)	10.834
Tongue		5.88	2.35E-09	90.01 (64.75-113.20)	9.41	89.15 (54.15-115.07)	10.28
Buccal	V4-5	−2.06	0.02	60.44 (25.5-140.08)	15.89	61.87 (29.13-95.52)	14
Saliva		−5.72	5.90E-09	61.18 (28.11-149.11)	16.82	63.68 (30.02-111.39)	14.61
Tongue		−4.39	6.27E-06	68.96 (34.18-144.62)	16.82	70.82 (35.96-99.01)	13.21

**Fig 3:**
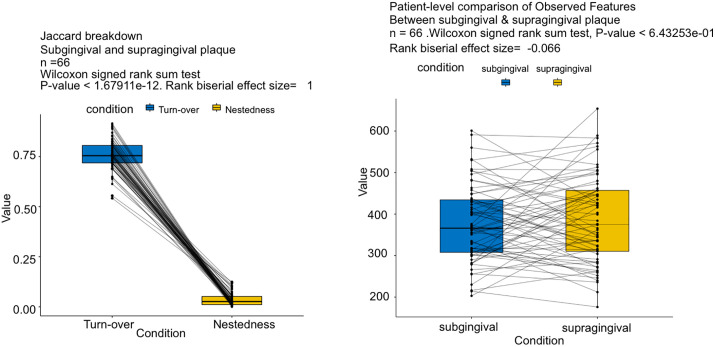
Breakdown of Jaccard dissimilarity. Fig 3A: Dissimilarity between supragingival and subgingival plaque in microbial membership within the same participant, illustrated through the two components that form the Jaccard dissimilarity; turnover, and nestedness. Fig 3B: Observed features alpha diversity between supragingival and subgingival plaque samples within the same individual.

Next, we analyzed the within-subject microbial dissimilarities across various oral niches to examine site-specific variations. FALAPhyl was run again on a new mapping file containing only paired samples of saliva, sub- and supra-gingival plaque at the baseline time point. The pipeline itself automatically excludes unwanted samples and runs the analysis without needing to pre-filter the biom count table. Paired-sample analysis (paired-alpha paired-beta paired-diff) was done. The observed ASV richness between supragingival and subgingival plaque was not significantly different (Wilcoxon signed-rank test, p > 0.05; Fig 3B).

Given that supragingival plaque is continuous with subgingival plaque, and is more readily exposed to saliva, we were interested in understanding how similar it is to saliva compared to subgingival plaque. To illustrate the ASV membership dissimilarity between sites, we used Jaccard dissimilarity. Jaccard dissimilarity analysis showed that saliva was equally dissimilar to both supragingival and subgingival plaque (Friedman’s test, FDR-adjusted p = 0.3). Therefore, we wanted to focus on the differences between supra- and sub-gingival plaque. Jaccard dissimilarity was broken down to its two components – turnover (replacement of an ASV by another) and nestedness (non-replacement leading to one site being a subset of the other) [18]. Dissimilarity decomposition revealed significantly greater turnover than nestedness between supragingival and subgingival plaque (Wilcoxon signed-rank test, p < 0.05; Fig 3A).This indicates that the missing ASVs in one site is replaced with one not found in the other site, so much so that the number of observed ASVs in the two was not significantly different from each other (p > 0.05).

By utilizing the internal reporting capabilities of Snakemake (snakemake include_biom_and_meta alpha beta breakdown diff network paired_beta paired_alpha paired_diff --report data/report.html), the original files, steps taken to generate the results, and the steps needed to generate them are packaged into a zipped file, thereby enabling provenance and transparency without locking the files into a specific format. This step also prevents erroneous attribution of analyses to incorrect pipeline runs (e.g.,: full samples vs only paired samples in this example) but packaging only the correct results in the report. This method also allows for custom analysis to be done as we have below, without needing to extract artefacts as is done with QIIME2.

We were interested in identifying the percentage of ASVs that were present in the one-week samples in the supra- and subgingival plaque samples that were not present in the child the week before, but were found in other family members, as well as those not linked to any familial sources. Reported as mean ± standard deviation (Table 2), the percentage of ASVs attributable to familial non-child sources was 0.169% ± 0.08%, which was smaller than those not attributable to any familial sources (0.548% ± 0.149%). However, the familial sources, represents a two-fold difference in the mean relative abundances compared to non-familial sources, (0.0206% ± 0.0126% and 0.0125% ± 0.0044%, respectively).

**Table 2:** Non-self sources of subgingival plaque using V1-3 region.

Source	ASV% (mean ± Standard deviation)	ASV Mean Relative Abundance % (mean ± standard deviation)
Familial, non-self sources	0.169% ± 0.08%	0.0206% ± 0.0126%
Non-familial, non-self sources	0.548% ± 0.149%	0.0125% ± 0.0044%

In summary, by using the default outputs of the two pipelines, an entire exploratory analysis can be done where insights can be gleaned into the data. In this example, the FAVABEAN provided indication of which ASV type was more appropriate to use to resemble taxonomic rank comparisons. Next, beta dissimilarity analysis with FALAPhyl showed that although saliva gets in contact with more supra-gingival plaque than subgingival plaque, saliva is equally dissimilar to both. Moreover, despite supra- and subgingival plaque are physically contiguous, the dissimilarly in membership is due to unique ASVs in each (turnover), rather than one niche having a subset of ASVs from the other. One week post-dental prophylaxis, the reconstituted subgingival plaque contained members of the bacteriome that were not from the participant themselves. Although a larger number of ASVs could not be linked to a known source, those that are non-self but from a family member occupied a larger portion of bacteriome abundance. This indicates that after dental prophylaxis, the contribution of novel ASVs to the subgingival microbiome is small.

### Case study 2: Fallow time in dental clinical settings

**Fig 4:**
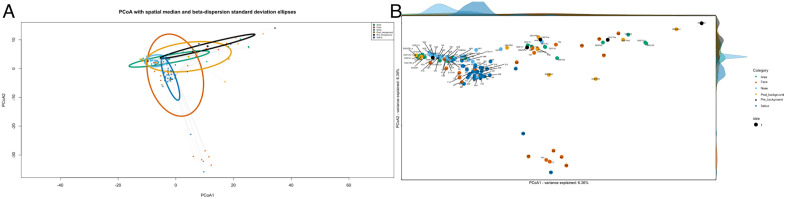
Principal Coordinates Analysis (PCoA) based on PhILR distances. **(A)** PCoA plots show dispersion using the standard deviation ellipses (representing one standard deviation of the spread of the samples away from the centroid, which may become cumbersome to illustrate any differences). **(B)** An alternate visualization of the same data using the probability density functions along principal axes, providing clearer distributional insight. The pipeline also generates Sample-labeled PCoA plots automatically.

The study was approved by the University of Alberta Research Ethics Board (Project No. Pro00120887). Methodology of the study has previously been published [25]. In summary, air samples were collected before and during various dental treatments and these samples were analyzed for the bacterial content to identify potential biological sources (nose, saliva) from the patients undergoing dental treatment. In this follow up analysis, we are using the pre-assembled ASVs of the full-length of the 16s rDNA provided by the sequencing facility in FALAPhyl, to examine the previously unanalyzed post-procedure air samples, in which their collection was done immediately after the end of the procedures and after evacuation of all patients and dental personnel, in which air sampling was done within 5 minutes of the end of the dental procedure. The objective is to compare the post-procedural air to pre- and intra-procedure air to assess for their microbial dissimilarity and inform infection control practices (Fig 4). Specifically, the goal was to determine whether the microbial composition of post-treatment air differed significantly from the baseline air, to assess the necessity of allowing time for the particles in the air to settle down (termed Fallow Time in Infection Prevention and Control disciplines).

Beta diversity analysis (group centroids, ADONIS) revealed no significant differences between post-procedural and pre-procedural air (ADONIS, FDR-adjusted p = 0.578; ANOSIM, FDR-adjusted p = 0.159), nor between post- and intra-procedural air (ADONIS, p = 0.49; ANOSIM, p = 0.32).

### Case study 3: Nitrate mouthrinse and microbiome stability

**Fig 5:**
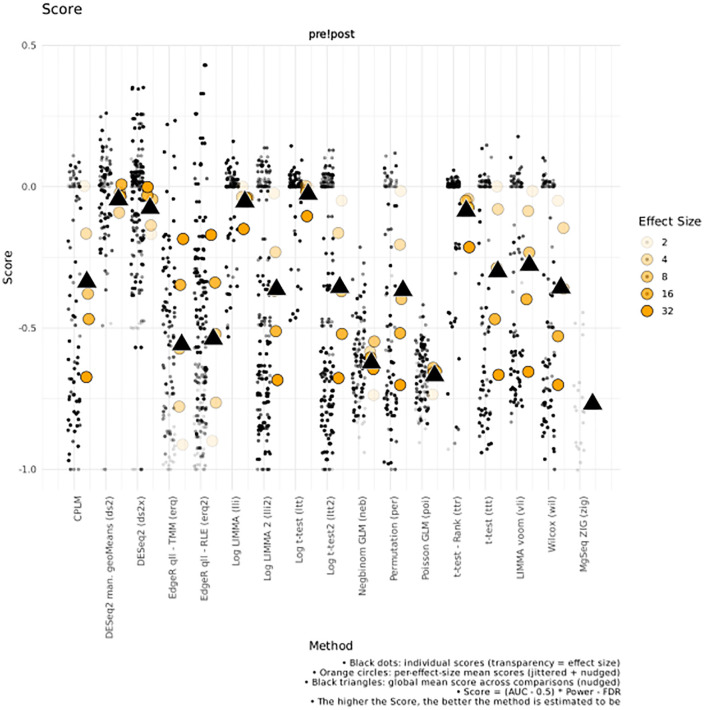
Scores from the paired testing in Intervention arm, baseline and post-intervention paired samples. The test shows the dataset interrogated against multiple spike-in trials, with different effect sizes. Black triangle illustrates the average of the effect sizes. As none of the averages reach the zero threshold, none of the methods are capable of reliably identifying the statistically significant features.

A parallel arm randomized clinical trial was conducted to test the effect of a nitrate-rich mouthrinses on the oral microbiome composition (details in Appendix 3 in S1 File). Study protocol review and ethical approval were obtained from the Dalhousie University Research Ethics Board (REB# 2024–7166). The study protocol and plan for analyses were registered as a clinical trial at ClinicalTrials.gov (identifier: NCT06588049) prior to recruitment and study commencement. Sequences are deposited in NIH SRA (project ID PRJNA1300299). In total, 21 participants were randomized to either the intervention group (n = 11; nitrate mouthrinse) or the placebo control group (n = 10) administered daily for 14 days. To enable species-level resolution, Sidle-reconstructed BIOM outputs from FAVABEAN (eHOMD v15.22) were used for downstream exploratory analysis. No significant differences in overall microbial composition were observed between intervention and placebo groups (ADONIS and ANOSIM; p > 0.05 for both tests). We next explored whether subtle species-level differences could be detected despite the absence of global shifts. Given the lack of a universally accepted method for differential abundance analysis — and the variability of results across methods — we employed the DAtest package to systematically assess test performance on this dataset [14]. DAtest package uses artificial spike-in simulations to empirically evaluate statistical test performance under varying conditions. Our pipeline integrates this approach to guide method selection based on dataset-specific resilience. Our modifications of the DAtest package (parallelization to the spiking tests, and custom graphing outputs with incorporation of an additional test) are detailed in Appendix 3 (S1 File). None of the paired statistical testing identified significantly differentially abundant species in either the intervention or the control groups (FDR adjusted p > 0.05 for all tests). Moreover, none of the methods had a score above zero, indicating that none of them can reliably identify the statistically significant features (Fig 5).

## Discussion

### Technical insights

FAVABEAN and FALAPhyl were developed to address key challenges in 16S rRNA amplicon data analysis including the need for automation, reproducibility and scalability across diverse computing environments. Although FAVABEAN is solely designed for amplicon-based sequencing workflows in mind, FALAPhyl’s analytical framework is data-agnostic and applicable to any compositional dataset, including those beyond 16S rRNA sequencing. The breadth of analytical tools created for FALAPhyl allow for both cross-sectional and paired study designs. This enables users to efficiently and comprehensively perform exploratory and hypothesis-generating analyses with minimal manual intervention. Together, FABABEAN and FALAPhyl demonstrate how containerization and workflow-based approaches can streamline complex bioinformatics analyses while maintaining transparency, version control and scientific rigor. The integration of Snakemake as the workflow engine for both pipelines provide several key advantages over traditional script-based analyses including scalability, reproducibility and automated provenance tracking. The automatic scaling from single computers to cloud implementations without the need for specialized infrastructure removes technical barriers that often limit researchers’ ability to process large datasets. By containerizing both pipelines, consistency across different computational environments eliminating issues related to software version discrepancies-a common source of irreproducibility in microbiome research [1].

A major benefit of using FAVABEAN is the automatic identification of the best parameters for trimming the data, and the choice of error tolerance. Both of these are crucial for reproducibility of DADA2 ASV production. Unfortunately, tutorials of DADA2 rely on analysing sequence quality drop at the 3’ end in relation to sequence length. This is done visually in images. Although FIGARO is recommended as an alternate method, the output of FIGARO still requires manual selection of the desired output. The pipeline makes the selection of parameters from FIGARO more easily, either retaining the most number of sequences, or the highest quality of sequences. Once a selection is made, the pipeline automatically proceeds with the downstream steps. In each step, the number of features retained is calculated and presented to the user. Moreover, since data analysis can be time consuming even within a pipeline, the pipeline uses Snakemake’s benchmarking features which automatically calculates disk space, RAM and CPU time usage, which allows the users to identify potential bottlenecks within the hardware. Furthermore, the integration of Sidle into the pipeline allows for samples sequenced with multiple primers to automatically gain resolution that is greater than seen from any of the single primer alone. This is done automatically and does not require following the manual database preparation steps needed that are suggested in their tutorial.

FALAPhyl’s integration of the DAtest framework addresses a critical challenge in the selection of a suitable differential abundance methods in the absence of a gold-standard approach. Although some differential abundance algorithms such as LefSe, ALDEx2, ANCOM, and DESeq2 have gained traction in recent years, the use of one instead of the other requires judicious and careful analysis, as they are dependent of the characteristics of the data itself [26]. A way to understand the data is to challenge the assumptions of the algorithms themselves through a spike-in challenge. These in-silico challenges simulate adding spikes to the data that are not correlated to the underlying relationships between the original features. An algorithm that identifies these spikes and maintains the relationships between the original features in the face of the spike challenges likely have assumptions that are more suitable to the data structure of the study. Many of the most popular differential abundance methods are implemented in the package and are easily identifiable and selectable as part of FALAPhyl’s yaml configuration file. The benefit of using these popular algorithms within the pipeline as opposed to directly on the dataset, however, tests the mathematical assumptions of these algorithms against the reality within the datasets, providing evidence of algorithm suitability. We enhanced DAtest within FALAPhyl by parallelizing spike-in simulations, which improves computational efficiency and enables its application to larger datasets. Moreover, the FALAPhyl’s automated handling of paired samples is particularly advantageous for longitudinal and intervention studies, where manual subsetting is error-prone and labor-intensive. Traditional approaches often require manual data subsetting and careful tracking of sample relationships, introducing opportunities for error. Automating these workflows reduces analytical burden and potential for user error, while Snakemake’s integrated reporting ensures complete provenance and reproducibility.

Recent years have seen the widespread adoption of several open-source workflows designed for reproducible 16S rRNA amplicon sequencing analysis. Arguably the most popular of methods is QIIME2, which has many but not all the features in our two pipelines. Its popularity is best illustrated of the breadth of plugins available for it, many of which provide similar results. This breadth of choice, however, puts the onus of the user to understand the output dependencies between the functions available in it. What these two pipelines lack in breadth makes up for with an opinionated collection of well-constructed, and well-integrated functions that can be used as part of an automated pipeline. This allows for s novel to have a powerful introduction into microbial bioinformatics, and for the expert user to rapidly explore the data prior to constructing custom analysis through code on the various intermediate file outputs. However, the opinionated collection of analyses in the two pipelines also provides some analyses not present in QIIME2, such as differential abundance spike testing, various decorations of beta diversity plotting that illustrate different features of the dataset, and teasing apart the two components that make up Jaccard dissimilarity. These features were carefully chosen due to their frequent use within our labs in the various projects prior to finally creating these two pipelines. However, in contrast to other popular pipelines, FAVABEAN and FALAPhyl are command-line only, with no graphical user interface (GUI) support (e.g., Galaxy, QIIME Studio). This is largerly due to the impetus for creating these pipelines, the impetus of creating FAVABEAN and FALAPhyl was to automate the most common analyses used within our respective academic research laboratories, rather than be built with the eye on broad community uptake. The two pipelines have been intentionally built with new graduate students in mind. After minimal command line training, analyses can be run automatically. The easiest path into utilizing the two pipelines is through container technology, which packaged all the dependencies in a ready-to-deploy environment. Since the code is available and is published in a permissible copyright notice, this does not preclude further additions and modifications for those interested in doing so. It also makes the pipeline more future proof: anyone can add either modify the current functions or add novel ones without relying on a plug-in system. This is one reason why we believe FAVABEAN and FALAPhyl fill a niche in the microbiome analysis landscape: they offer advanced functionalities within a reproducible, Snakemake-based framework. These features particularly benefit researchers seeking flexibility, statistical rigor, and reproducibility.

### Biological insights from case studies

In Case study 1, we assessed microbial similarity across family members and oral niches. While second-generation sequencing has massively lowered the cost of DNA sequencing compared to that of Sanger sequencing, It sacrifices sequence read-length to compensate for parallelization of sequencing. This serves as a challenge; Sanger sequencing can sequence the much longer stretches of the 16s rDNA gene, which provides excellent identification capability of the taxonomy through coverage of the regions that contain the characteristic mutations of these taxa. This was not the case for second-generation sequencing technologies such as Illumina sequencing. Therefore, alternate strategies, such reconstruction, were employed to provide the similar breadth of taxonomic coverage and sample composition. The development of ASVs allowed for more granular analysis at the genome level, however that presents a challenge on how to integrate the granularity found in one primer into another. This case study shows the strength of both pipelines. Both per-amplicon region analyses and reconstructed Sidle features can be compared and contrasted, with both graphical and statistical illustrations being automatically generated. Then, in addition to the features expected in most popular pipelines such as beta diversity pairwise matrix calculations, popular dissimilarity methods such as Jaccard contain valuable information (its breakdown into its two components) that is generally not exposed within the popular pipelines. This allows for more granular understanding of how alpha diversity (feature presence) and their beta diversity patterns (turnover versus nestedness) can be understood together. Due to the direct availability of the intermediate data such as per-group beta diversity analyses without them being packages in a format protective of provenance, one can easily glean into the data and construct analyses not present in the pipeline. For example, tab-separated versions of the BIOM files are generated which allowed us to understand the presence and absence of non-self familial and non-familial seeding of ASVs. Although seeding not a novel finding onto itself, the novelty is in the fact that such seeding was detectable at all. To our knowledge, this is the first study to quantify the contribution of non-host familial microbiomes to recolonization of the subgingival plaque niche. The majority of previous studies have concentrated on maternal/child relationships, rather than whole family transmissions [27]. As similarity in the oral microbiota between the mother-child dyad increases during childhood and adolescence [27], our results provide quantitative evidence of such seeding. Oral prophylaxis is used to remove the supragingival plaque of the child as mean to “professionally” brush teeth. Supragingival plaque debridement, and especially professional debridement, has long been known to have an effect on the subgingival clinical status, and its microbiome [27–35], with the effect likely negatively associated with the increasing depth of the periodontal pocket. This is the first time that seeding from outside sources of the person’s the oral cavity be attributed to the contribution of the restitution of the subgingival microbiome. While we followed the participants for only one week, it is possible that further maturation of the biofilm would reduce the external contributions as the resident microbiota regain control over their niches. We suspect this is only partially true as dispersion events occur daily through tooth brushing, which has been shown to enhance the microbial similarity between twins [36]. Therefore, we believe regular oral hygiene likely increases the acquisition from external sources, through a process of repeated dispersal events. Familial ASV transmission was approximately twice that of non-familial sources, warranting further investigation into close-contact microbial exchange within households.

Microscopic examinations have shown the supra and sub gingival plaque are anatomically contiguous, allowing for continuous microbial exchange between these niches. The gingival crevicular fluid carries planktonic-state bacteria, as well as flecks of biofilm from the subgingival environment toward the oral cavity, thereby meeting the supragingival plaque. This provides avenues of microbial dispersal between the two environments. Dissimilarity decomposition revealed that inter-site variation was driven primarily by species turnover rather than nestedness, suggesting that each site harbors distinct microbial communities adapted to its environment. Therefore, one should not assume that ecological filtering by way of changes in the environment such as reduced Oxygen tension, or changes in the nutritional resources in the environment, caused attrition in the microbial composition resulting in their differences. Rather, the driver of the differences between the two is largely due to the distinct membership in the two sites, likely adapted to exploit the ecological differences in the two sites. This was possible to understand due to the use of betapart package, which breaks Jaccard into its two components. For those interested in extending the pipeline, one can look at how a CRAN package such as betapart is integrated into the pipeline and build upon that using their most used packages.

In this case study, the primer‑averaging module of FAVABEAN enabled seamless consolidation of V1–V3 and V4–V5 amplicon data, reducing primer-driven bias in downstream taxonomic inference. Simultaneously, FALAPhyl’s automatic paired‑sample analysis allowed within-subject beta diversity and differential abundance testing without preprocessing steps, preserving analysis provenance and minimizing oversight. These integrated features facilitated clear, reproducible assessments of family microbial similarity that would be cumbersome in manual multi-primer workflows.

#### In Case study 2 we assessed the need for fallow time in dental clinical settings.

Fallow time, introduced during the COVID-19 pandemic, represented a logistical challenge by delaying post-patient practice turnover to allow for aerosol particles to settle to the floor. Our analysis showed that microbial composition in post-procedural air was statistically indistinguishable from pre-procedural air samples. This finding has practical implications for infection control protocols in dental practice. Aerosolized bacteria either settle or disperse rapidly, potentially reducing the need for extended fallow times between patients, or that relying on the bacterial content of the air from known sources as a surrogate to air contamination is not a good surrogate to actual quantitative measurement of the microbial agent in the air. Interpretation of these findings warrants caution. Larger studies incorporating quantitative pathogen detection methods (e.g., qPCR or culture-based assays) are needed to validate microbial risk assessments based solely on amplicon sequencing data.

In this case study, FALAPhyl’s automated beta diversity generation and paired-group testing demonstrated that post-procedural air microbial composition was statistically indistinct from pre-procedural levels. These detailed comparisons — powered by Snakemake-driven provenance, PhILR distance calculations, and ADONIS/ANOSIM tests — provide timely, reproducible evidence relevant to infection control decisions, and could be replicated or extended with minimal reconfiguration.

#### In case study 3 we assessed the efficacy of a nitrate-rich mouthrinse on oral microbiome stability.

The absence of detectable microbiome shifts following the use of the nitrate mouthrinse highlights the resilience of oral microbiome. Despite nitrate’s known role in promoting nitrate-reducing bacteria and potentially influencing oral and systemic health through the nitrate-nitrite-nitric oxide pathway, we observed no compositional shifts after 14 days of daily use. This stability likely reflects several biological factors: the tongue microbiome’s inherent resilience in healthy individuals [23], the possible inadequacy of once-daily dosing to exert sustained selective pressure, and the short intervention period relative to the stability of established microbial communities. These findings align with emerging evidence that healthy oral microbiomes resist perturbation from single-agent interventions [37]. Unlike the disruption seen with broad-spectrum antimicrobials, a metabolic substrate like nitrate may require either longer exposure, higher frequency of use, or a dysbiotic baseline state to induce measurable shifts in the community composition. This is consistent with the *Anna Karenina’s principle* where dysbiotic microbiomes exhibit greater variability compared to stable, more homogenous healthy profiles [38]. The participants’ healthy periodontal likely contributed to this observed microbial stability, as diverse and balanced communities typically demonstrate greater resilience to external perturbations [37]. These results suggest that microbiome-targeted interventions in healthy populations may need to consider the ecological stability of established communities. Future studies should consider recruiting individuals with existing dysbiosis and extending intervention duration to evaluate whether metabolic shifts precede detectable compositional changes.

This case study highlights how FALAPhyl incorporated DAtest-based empirical evaluation of several differential abundance methods using dataset‑specific spike-in simulations. This guided selection of appropriate tests and validated the null finding of no significant microbiome shift, reinforcing confidence in the result’s validity. Without this framework, researchers would lack both systematic test selection and documentation of method suitability.

### Limitations and future directions

Several limitations of the current pipeline and its implementation merit discussion.

First, FAVABEAN currently supports only paired-end sequencing, which limits its applicability to studies using single-end read formats—a common approach in some targeted amplicon studies.

Second, while many steps in both pipelines are automated, users are still required to specify key analysis parameters such as diversity metrics and distance measures. Although sensible defaults and detailed documentation are provided, a basic understanding of bioinformatics and microbiome statistics remains necessary to ensure optimal and accurate usage.

Third, while the pipelines are well-suited for standard 16S workflows, novel or highly specialized analyses (e.g., rarefaction-free methods, phylogenetic network models) would require customization beyond the current implementation. The modular nature of Snakemake facilitates such extensions; however, doing so demands some programming proficiency.

Additionally, reliance on Docker containerization, though essential for reproducibility and version control, may pose access limitations for users operating in high-security or restricted computing environments where container technologies are not supported.

Despite these limitations, the open-source design of FAVABEAN and FALAPhyl promotes community-driven development, adaptation, and extension, enabling researchers to tailor the workflows to evolving needs and diverse experimental designs.

## Supporting information

S1 File Appendices. Appendix 1 – FAVABEAN and FALAPhyl detailed descriptions of the functions and how-to manual. Appendix 2 and 3 are detailed materials and methods of the case studies.
(PDF)
