# Supplementary material for: FAVABEAN and FALAPhyl: Open-source pipelines for scalable 16s rRNA microbiome data processing and visualization
Source: PLoS One. 2026 Apr 7;21(4):e0331145. doi: 10.1371/journal.pone.0331145 (PMC13056196; doi:10.1371/journal.pone.0331145)
Supplement: S1 File — Appendix 1 – FAVABEAN and FALAPhyl detailed descriptions of the functions and how-to manual. Appendix 2 and 3 are detailed materials and methods of the case studies. (PDF) [file pone.0331145.s001.pdf]

Appendix 1

Detailed supplementary

FAVABEAN

Part 1: study set up

The following steps illustrate using FAVABEAN using Docker in an interactive mode.

```
➢ docker run --rm -it -v Path/To/Local/Folder:/data
  khalidtab/favabean:latest start
```

Although the image contains both bash and sh, running the pipeline with the `start` will print commands needed to use the pipeline, as below:

```
Copying the template files in your directly if they don't
exist (environments.txt, files_info.csv, and favabean.yaml)'
If this is the first time you are running the pipeline on this
dataset, you will need to initialize your 'files_info.csv'
file.
Please write 'conda activate' and copy the biom.yaml
environment link from FAVABEAN_environments.txt file

Example:
 conda activate <<biom environment such as :
.snakemake/conda/007a7beaa3353a33c938a5a0e57be4ff_>>

Then, execute the following command
 Rscript --vanilla workflow/scripts/batch.R


Example command(s):
snakemake paired_taxonomy --use-conda --cores all --keep-going
--retries 5 --rerun-incomplete --scheduler greedy
Please copy and paste the above command(s) or type your own
command below.
```

Below is the step-by-step tutorial on using the pipeline

1. Create the metadata table for the sequencing FASTQ files

As mentioned in the prompt above, when `start` was executed, files_info.csv was copied to your `Path/To/Local/Folder`. This comma-separated table is where the fastq files are identified. Below is a minimal example:

sample	fastq1	fastq2	primer_5	primer_3	region	Batch_ID	expected.length
P10A1	P10A1_27F_S1482_L001_R1_001.fastq.gz	P10A1_27F_S1482_L001_R2_001.fastq.gz	ACACTCTTTC	GTGACTGGA	F27_R519		500
P10A1	P10A1_27F_S3201_L001_R1_001.fastq.gz	P10A1_27F_S3201_L001_R2_001.fastq.gz	ACACTCTTTC	GTGACTGGA	F27_R519		500
P10A1	P10A1_V3V5_S2229_L001_R1_001.fastq.gz	P10A1_V3V5_S2229_L001_R2_001.fastq.gz	ACACTCTTTC	GTGACTGGA	F515b_926R		500
P10A1	P10A1_V3V5_S510_L001_R1_001.fastq.gz	P10A1_V3V5_S510_L001_R2_001.fastq.gz	ACACTCTTTC	GTGACTGGA	F515b_926R		500
P10A2	P10A2_27F_S1512_L001_R1_001.fastq.gz	P10A2_27F_S1512_L001_R2_001.fastq.gz	ACACTCTTTC	GTGACTGGA	F27_R519		500
P10A2	P10A2_27F_S3231_L001_R1_001.fastq.gz	P10A2_27F_S3231_L001_R2_001.fastq.gz	ACACTCTTTC	GTGACTGGA	F27_R519		500
P10A2	P10A2_V3V5_S2260_L001_R1_001.fastq.gz	P10A2_V3V5_S2260_L001_R2_001.fastq.gz	ACACTCTTTC	GTGACTGGA	F515b_926R		500
P10A2	P10A2_V3V5_S541_L001_R1_001.fastq.gz	P10A2_V3V5_S541_L001_R2_001.fastq.gz	ACACTCTTTC	GTGACTGGA	F515b_926R		500

Columns:

- **sample**: This is where you note the eventual name of the samples in the biom file. Note that in this example, we have the same sample, identified by the first column, sequenced multiple times, using two different primers. The merging strategy will be discussed in later stages of this tutorial.
- **fastq1, fastq2**: path to the fastq files in your `Path/To/Local/Folder`
- **primer_5, primer_3**: the forward and reverse primers used for producing the amplicons.
- **region:** name of the sequenced region. This is used as a placeholder for the primer tables. Any name to identify the region is fine.
- **Batch_ID:** see below

- **Expected_length:** expected length of the amplicon. If unknown, the largest length is acceptable. Generally speaking, Illumina MiSeq chemistry allows for 350 bp in both forward and reverse reads. After primer sequences removal, a 500 bp length is reasonable and therefore used as default.

2. (Optional) Identify sequence batches

As the machine learning algorithm Error Learning Algorithm of DADA2(3) is batch-specific, we need to identify the batch of each sample. Failure to explicitly identify the batches will likely degrade the quality of the error learning. If this information is not available, the following step is executed.

```
➢ conda activate <<biom environment such as :
  .snakemake/conda/007a7beaa3353a33c938a5a0e57be4ff_,
  environment path can be retrieved from the
  environments.txt file>>
```

Then execute the following command

```
➢ Rscript --vanilla workflow/scripts/batch.R
```

This will read the headers of each of the files that are in the Illumina CASAVA 1.8+ format, and will segment the files based on the following schema:

@<instrument>:<run number>:<flowcell ID>:<lane>…

Once successfully executed, a new CSV file, file_info_Batches.csv, is generated with the correct information as below:

sample	fastq1	fastq2	primer_5	primer_3	region	Batch_ID	expected.length	alias	SampleNum
P10A1	P10A1_27F_S1482_L001_R1_001.fastq.gz	P10A1_27F_S1482_L001_R2_001.fastq.gz	ACACTCTTTC	GTGACTGGA	27F519RRvR	Batch2	500	P10A1	6
P10A1_1	P10A1_27F_S3201_L001_R1_001.fastq.gz	P10A1_27F_S3201_L001_R2_001.fastq.gz	ACACTCTTTC	GTGACTGGA	27F519RRvR	Batch3	500	P10A1	7
P10A1_2	P10A1_V3V5_S2229_L001_R1_001.fastq.gz	P10A1_V3V5_S2229_L001_R2_001.fastq.gz	ACACTCTTTC	GTGACTGGA	515bFTruSeq	Batch3	500	P10A1	8
P10A1_3	P10A1_V3V5_S510_L001_R1_001.fastq.gz	P10A1_V3V5_S510_L001_R2_001.fastq.gz	ACACTCTTTC	GTGACTGGA	515bFTruSeq	Batch2	500	P10A1	9
P10A2	P10A2_27F_S1512_L001_R1_001.fastq.gz	P10A2_27F_S1512_L001_R2_001.fastq.gz	ACACTCTTTC	GTGACTGGA	27F519RRvR	Batch2	500	P10A2	10
P10A2_1	P10A2_27F_S3231_L001_R1_001.fastq.gz	P10A2_27F_S3231_L001_R2_001.fastq.gz	ACACTCTTTC	GTGACTGGA	27F519RRvR	Batch3	500	P10A2	11
P10A2_2	P10A2_V3V5_S2260_L001_R1_001.fastq.gz	P10A2_V3V5_S2260_L001_R2_001.fastq.gz	ACACTCTTTC	GTGACTGGA	515bFTruSeq	Batch3	500	P10A2	12
P10A2_3	P10A2_V3V5_S541_L001_R1_001.fastq.gz	P10A2_V3V5_S541_L001_R2_001.fastq.gz	ACACTCTTTC	GTGACTGGA	515bFTruSeq	Batch2	500	P10A2	13

3. Select the parameters for FAVABEAN

The parameters are available in the favabean.yaml file. Deselecting a parameter is simply done by commenting out the beginning of the file with “#”. Below are options:

- **initial_filter**
    - This step helps remove sequences not expected to give you a meaningful hit to DADA2 because they are too short.
- **trim_param**
    - Automatic identification of the trimming parameters for DADA2 typically requires manual work, which may lead to reproducibility errors. This pipeline uses Figaro for this step. Figaro expects all sequences to be of the same length, so cutting all sequences to a specific length is needed for this step. These trimmed sequences are only used in Figaro and are not used during the other steps. This trimming also simplifies the Figaro analyses, and it helps

- You can write a specific number you like (eg 100, for 100 basepairs), or, you can filter out sequences based on general parameters as below
    - Default: is that if the length of sequences in Q1 is within 10% of Q2 (median), then use the Q1 to include the majority of sequences. Otherwise Q2 is used
    - Q1: 25th percentile length
    - Q2: 50th percentile length (median)
    - Q3: 75th percentile length
    - max_len: longest sequence length
- Note that this accepts one value only. So if you have more than 1 uncommented, only the first one will be used.

➢ **Figaro(20)**

- Figaro provides multiple options for the DADA2 parameters along with their error quality scores. The pipeline is implemented with the option of the two extremes:
    - highest_coverage: this option retains the largest percentage of sequences
    - lowest_errors: this option uses the parameters with the largest score.

➢ **taxonomy_database**

- Bacterial identification is done through DADA2. By default, the links to two databases are provided; eHOMD(34) and SILVA(35). If the path to the files is not available in your `Path/To/Local/Folder`, the files will be downloaded for the `Path/To/Local/Folder/resources`. Adding new

reference databases is trivial by either identifying their downloading link in the yaml file, or by creating a placeholder entry in the yaml file, and placing the reference databases in the `resources` folder.

3. Execute the pipeline

```
snakemake paired_taxonomy --use-conda --cores all --keep-going
--retries 5 --rerun-incomplete --scheduler greedy
```

Note: Currently, only analysis of paired fastq files are implemented. Future versions will work on forward sequenced fastq files.

Two options available for analysis: `paired_taxonomy`, and `paired`. The difference between the two is that `paired_taxonomy` will identify the taxonomy of the sequences, in addition to generating the ASVs that `paired` does. Below are explanations of SnakeMake options, not specific to the pipeline.

- `--use-conda:` This is mandatory for the proper execution of the pipeline
- `--cores:` Either a numeric value is to be provided, or `all` for all cores available to the docker container.
- (optional) `--keep-going:` In case of an error, all non-dependent branches of the analysis will continue their execution.
- (optional) `--rerun-incomplete:` in case of an error, rerun the step
- (optional) `--retries:` in case of an error, retry the step X number of times.

- (optional) `--scheduler:` options are `greedy,` and `ilp.` The latter option tries to reduce runtime and disk usage by best possible use of resources, but in some cases, takes a long time to find the best path for scheduling the steps.

Part 2: Pipeline steps explanation

Below is the graphic representation pipeline, with explanation of each step.

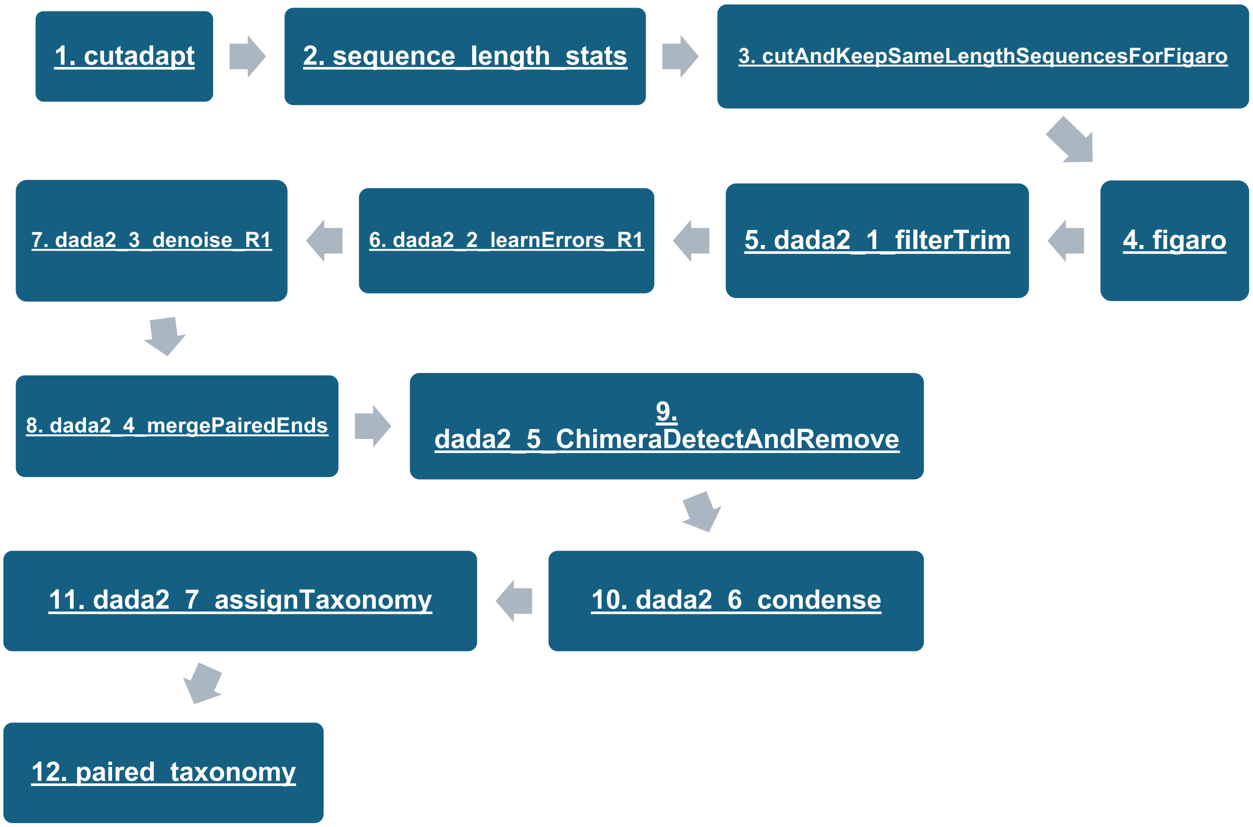

- **1. cutadapt**

- Removes adaptors from sequences based on values in files_info_Batches.csv

- **2. sequence_length_stats**
  - Calculate descriptive statistics using the package SeqKit on measure the length of the forward and reverse sequences

- **3. cutAndKeepSameLengthSequencesForFigaro**
  - Using SeqKit, the script will filter the FASTQ files based on the descriptive statistics from the step before

- **4. figaro**
  - Calculates the optimum trimming parameters for DADA2

- **5. dada2_1_filterTrim**
  - Using the output from step 1, filtering is done based on parameters provided by step 4

- **6. dada2_2_learnErrors_R1**
  - Learns sequencing errors on forward reads
  - Note: dada2_2_learnErrors_R2 is run in parallel for reverse reads

- **7. dada2_3_denoise_R1**

- Creates the model for denoising of forward reads
    - Note: dada2_3_denoise_R2 is run in parallel for reverse reads

- **8. dada2_4_mergePairedEnds**
  - Both forward and reverse denoised reads are reads and merged

- **9. dada2_5_ChimeraDetectAndRemove**
  - Chimeras are detected and removed

- **10. dada2_6_condense**
  - In-house developed faster version of DADA2_COLLAPSE_NOMISMATCH function. DADA2 version loops through all sequences one by one and determines whether they are “collapsable”. The optimized version precomputes the prefixes, and groups them by these prefixes prior to the loop that determines if these groups are collapsable.

- **11. dada2_7_assignTaxonomy**
  - Assigns taxonomy based on the selected database reference

- **12. paired_taxonomy**
  - If two primers exist for each sample, then primer averaging is performed

FALAPhyl

Part 2: Pipeline steps explanation

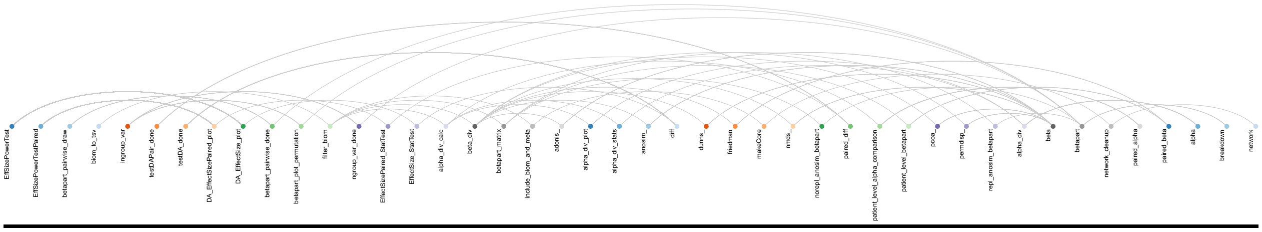

Above is the graphic representation of the steps, and their inter-connectedness. Due to its complexity, we will focus on the endpoints of the pipeline as accessible through the command line to run them all:

```
➢ snakemake alpha beta breakdown diff network paired_beta
  paired_alpha paired_diff --use-conda --cores all --keep-going
  --retries 5 --rerun-incomplete --scheduler greedy
```

Below, any variables defined in the falaphyl.yaml file that are used in the explanation are written inside square brackets “[variable name]”. The graphs are provided as they would be generated through the algorithm using the generic sizes that are written by default in the falaphyl.yaml file, but they may be adjusted as needed.

`alpha`: this command generates the following files

- *alpha_div/calc_[mysample]–[alpha].txt*
  - The raw calculations of alpha diversity per sample

- *alpha_div/stats_[mysample]–[alpha].txt*
  - Kruskal-Wallis test as a omnibus test, with post-hoc pairwise group tests using Wilcoxon rank-sum test with continuity correction with FDR

- *Plots/alpha_div_[mysample]/[group]–[alpha].svg*
  - Violin plots with only statistically significant comparisons indicated (Wilcoxon rank-sum test).

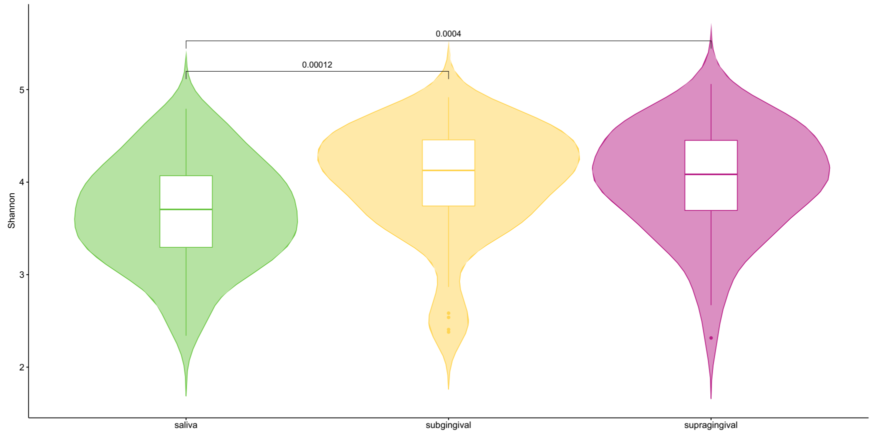

`beta:` this command generates the following files:

The raw calculations of beta diversity dissimilarity

- *distance/beta_div/ [mysample]–[distances].txt*
    - The raw calculations of beta diversity dissimilarity

ANOSIM (ANalysis of SIMilarity) outputs

- *distance/ANOSIM/[mysample]/anosim–[distances]–[group].txt*
    - Omnibus test
- *distance/ANOSIM/[mysample]/anosim–[distances]–[group]_pairwise.txt*
    - ANOSIM as a post-hoc test for pairwise comparison with FDR-adjusted p-values

ADONIS (Permutational Multivariate Analysis of Variance using distance matrices using adonis2 vegan implementation) outputs

- *distance/ADONIS/[mysample]/adonis–[distances]–[group].txt*
    - Omnibus test
- *distance/ADONIS/[mysample]/anosim–[distances]–[group]_pairwise.txt*
    - ADONIS as a post-hoc test for pairwise comparison with FDR-adjusted p-values

Beta dispersion (Multivariate homogeneity of groups dispersions (variances))

- *distance/PERMDISP/[mysample]/betadisper–[distances]–[group].txt*
    - Both the omnibus-test, and the pairwise analysis

Dunn's test for multiple comparisons

- *distance/PerGroupDistCompare/[mysample]/Dunns–[distances]–[group].txt*
    - Comparison of the distances per group-pairs.
    - Used to test if the distances between three groups are statistically different from each other or not.
        - Eg: saliva-subgingival plaque compared to saliva-supragingival plaque tests if the distance between saliva and the subgingival plaque is statistically different from that from saliva to supragingival plaque

Plots

Beta dispersion through Principal Coordinates Analysis (PCoA)

- *plots/PCoA_betadispersion–[mysample]–[distances]–[group].svg*
    - The ellipsoids are drawn with 1 standard deviation of the dispersion, as per Vegan's default.

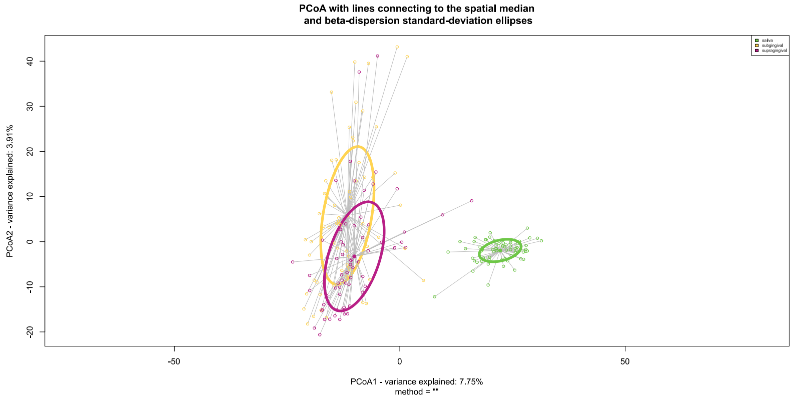

- *plots/boxplot_betadispersion–[mysample]–[distances]–[group].svg*
    - Box-plots of the beta dispersion away from the centroids.

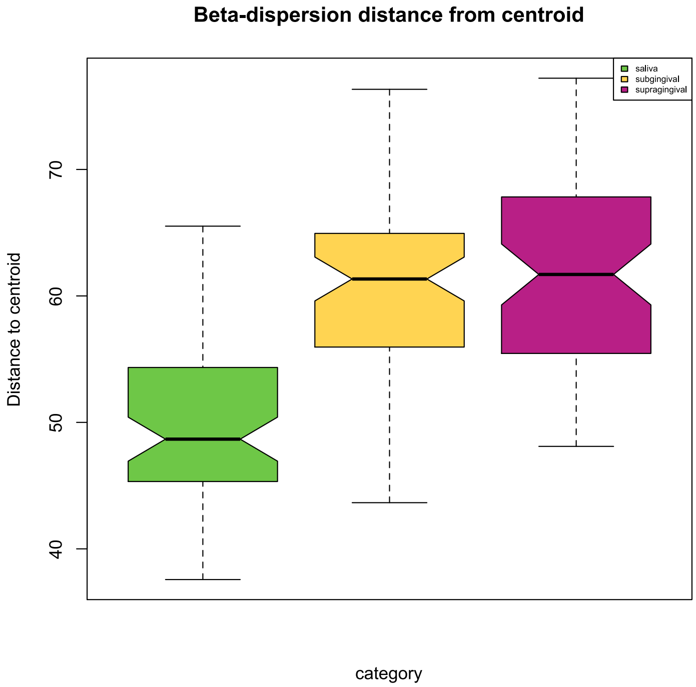

Principal Coordinates Analysis (PCoA)

In addition to the PCoA with the beta dispersion above, four different versions of PCoA plots are also generated, depending on the presence/absence of two decorations; probability density plots, and sample names. Plots are as below:

- *plots/betaDiv_[mysample]/PCoA–[distances]–[group].svg*

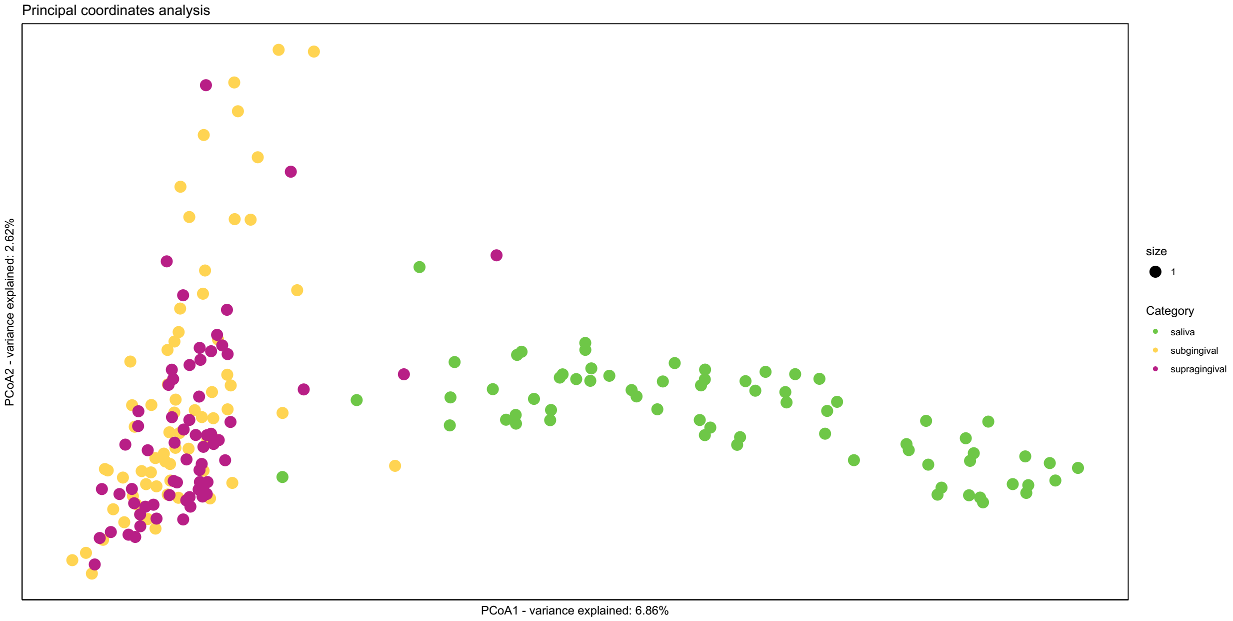

- *plots/betaDiv_[mysample]/PCoA–[distances]–[group].svgnoNameswProbDF.svg*
    - Same as the above plot but with probability density functions on the PC1 and PC2 plots. These can be useful when you have 4+ groups being plotted with overlapping dispersion that understanding the distribution of the groups, as seen in the example below

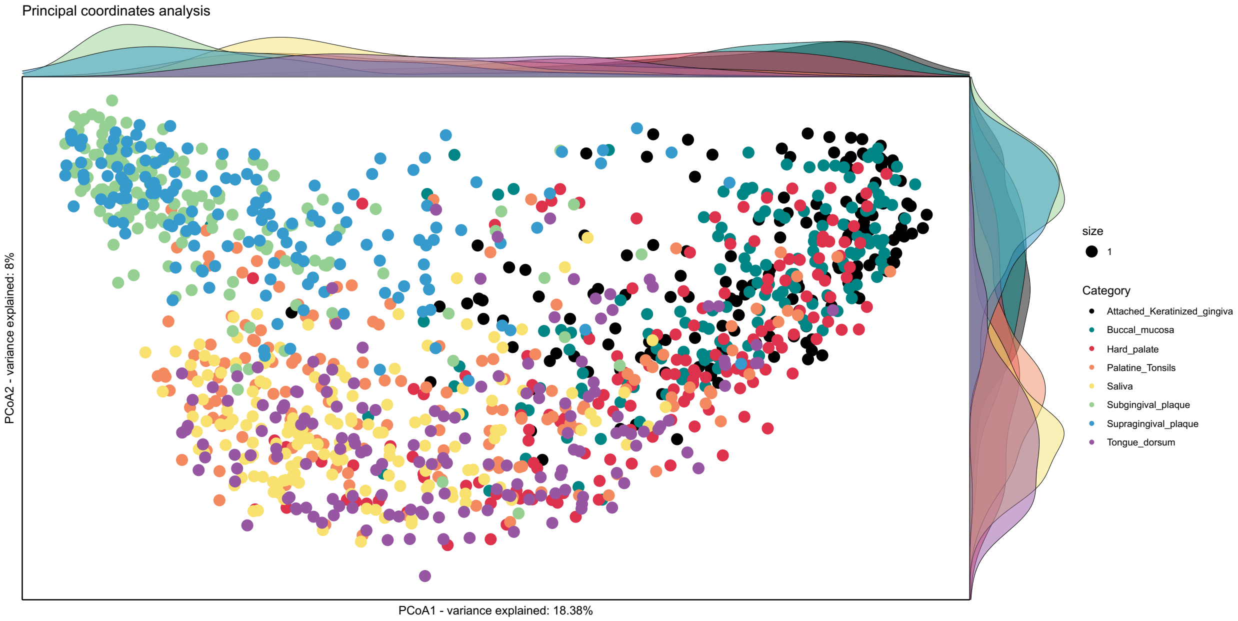

- *plots/betaDiv_[mysample]/PCoA–[distances]–[group].svgwithnames.svg*
    - PCoA graph, with sample names. Some overlapping of names is allowed as you can see below.

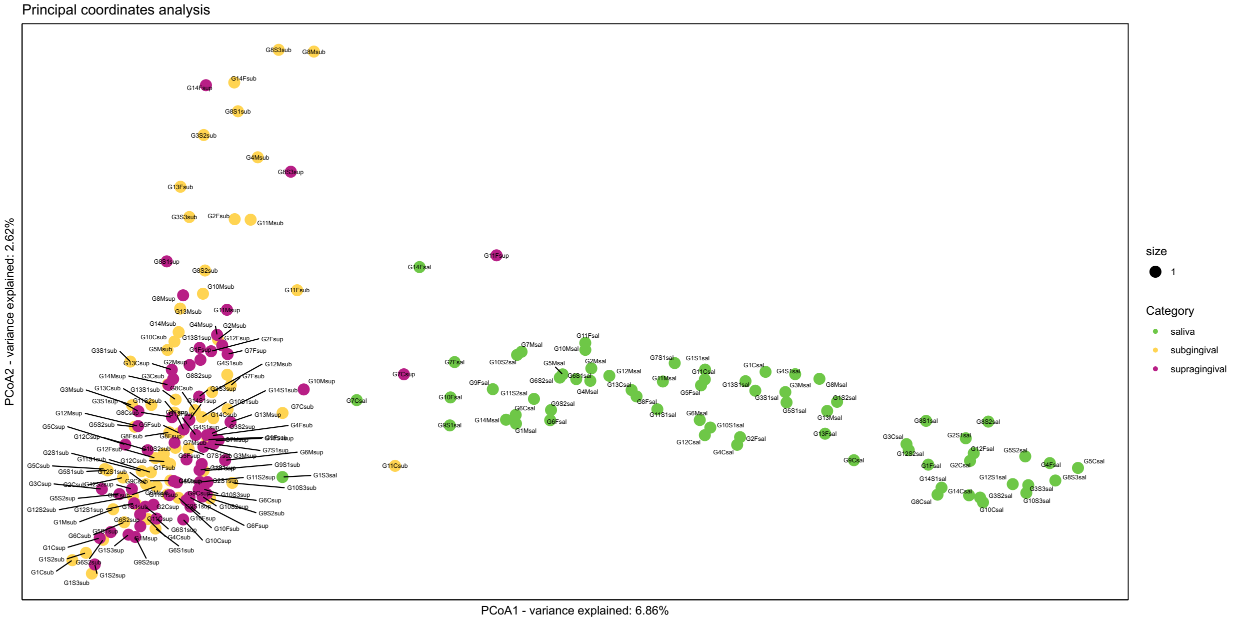

- *plots/betaDiv_[mysample]/PCoA–[distances]–[group].svgwithnamesprobDF.svg*

- ○ Combination of the two decorations above (PDF and sample names)

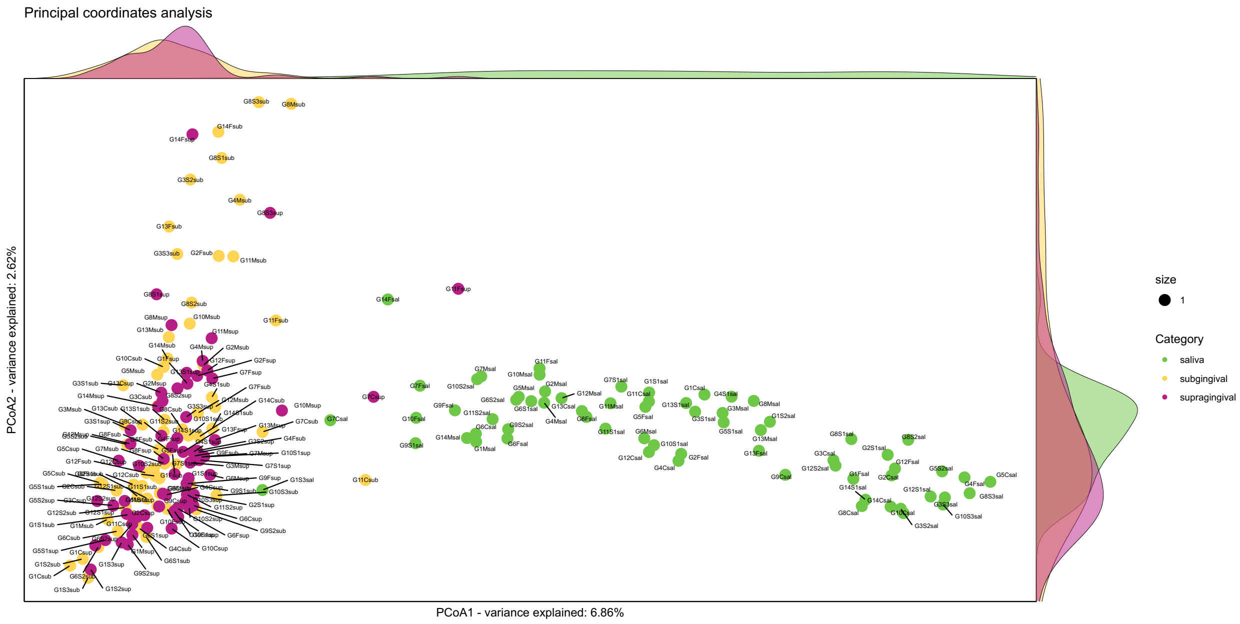

Non-metric Dimensionality Scaling (NMDS)

The four plots above are also available as NMDS plots.

- *plots/betaDiv_[mysample]/NMDS–[distances]–[group].svg*

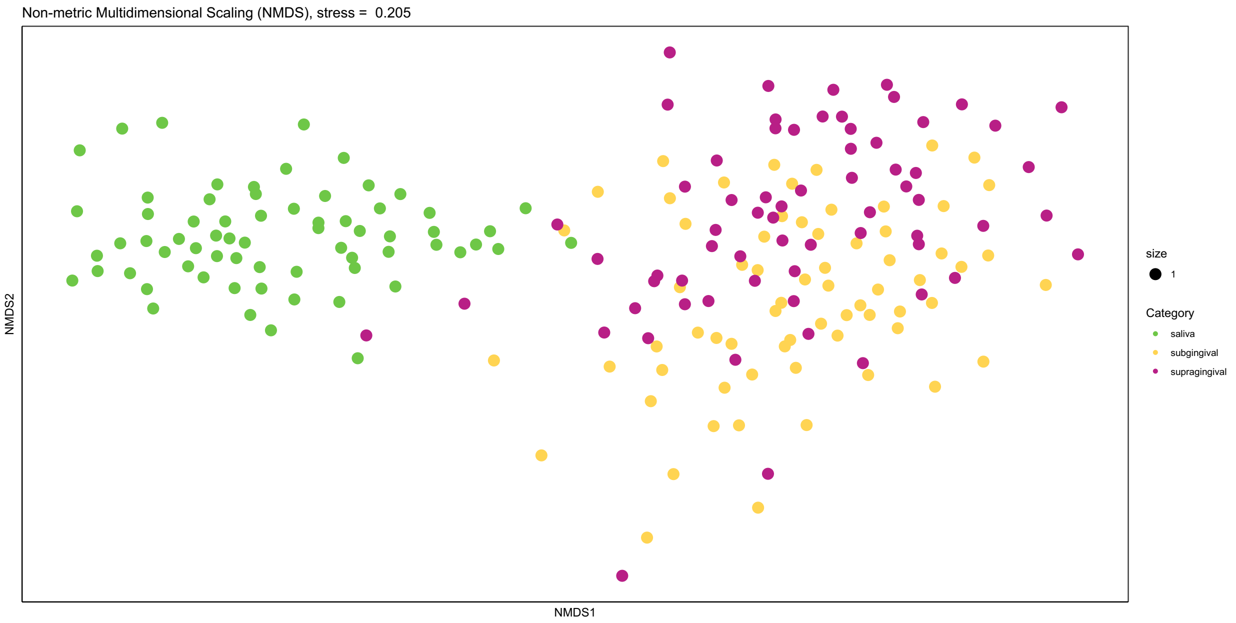

- *plots/betaDiv_[mysample]/NMDS–[distances]–[group].svgnoNameswProbDF.svg*

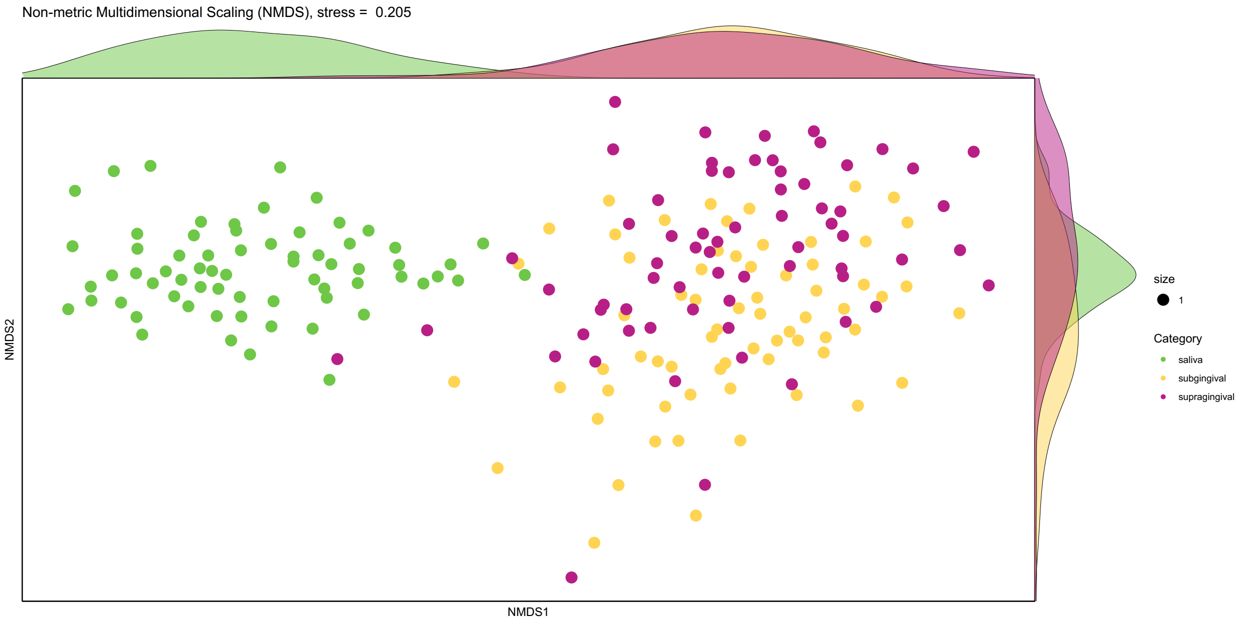

- *plots/betaDiv_[mysample]/NMDS–[distances]–[group].svgwithnames.svg*

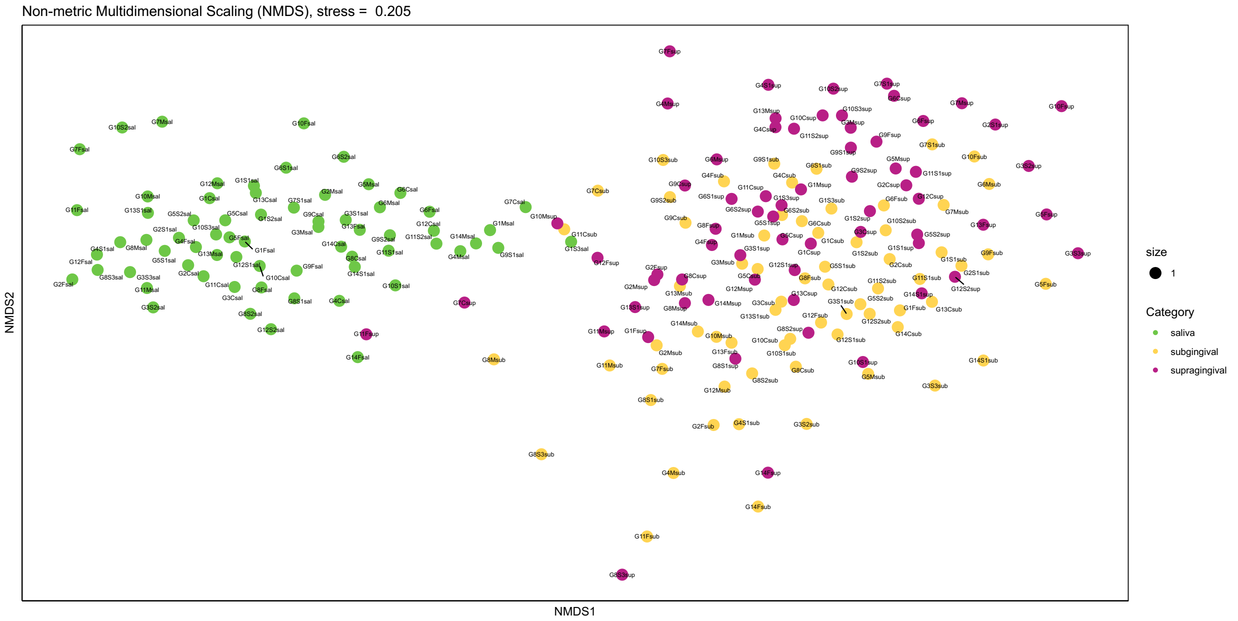

- *plots/betaDiv_[mysample]/NMDS–[distances]–[group].svgwithnamesprobDF.svg*

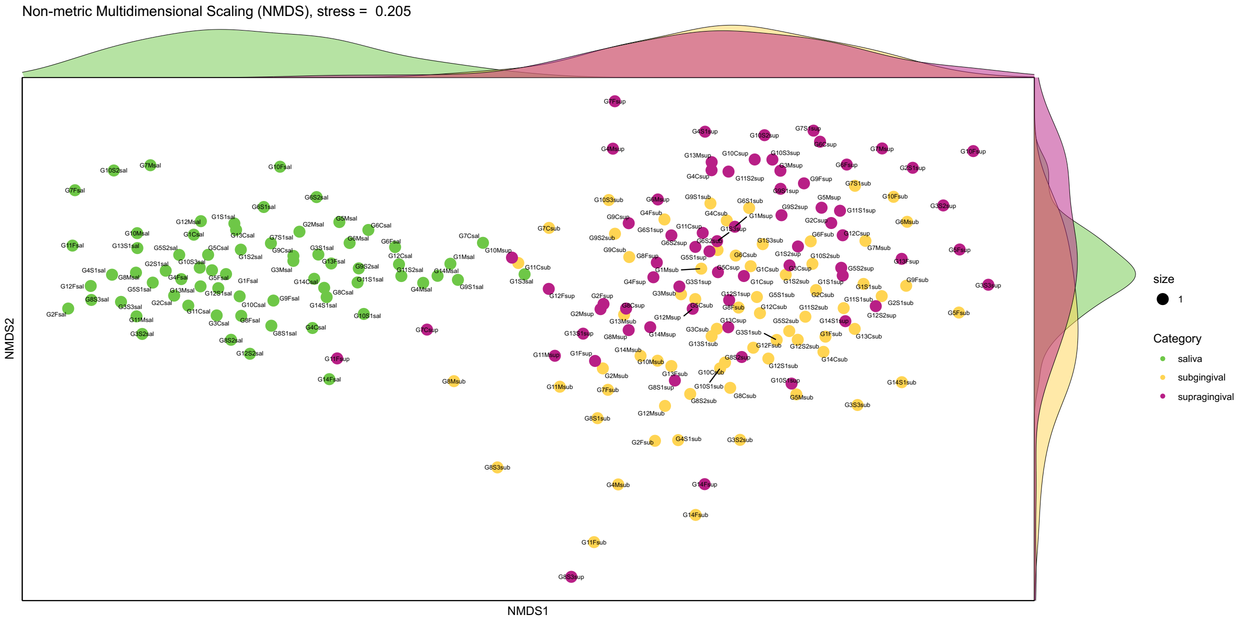

`breakdown`

This commands is based on Baselga's work on breaking down two popular dissimilarity matrices; Bray-Curtis, and Jaccard dissimilarities, between two sites into two components; features that are replaced and those that are not replaced resulting in one site being a superset of another. The `breakdown` command analyzes pairwise samples into these two components. In this command, [distances] is either bray for Bray-Curtis dissimilarity, or jaccard for Jaccard dissimilarity. Generated files are as follows:

- *betapart/[mysample]–[distances]–[category]/ingroupdiff/[category]+[group:group1]-ingroupdiff.txt*
    - Results of within-group differences between samples.
    - Each category in [group] is generated separately in this folder
    - Within-group differences are reported as
        - Dissimilarity: raw values, and percentages of the total dissimilarity
        - Permutation based mean values: both means, and standard deviations of the distribution are calculated for dissimilarity, and its two components. These are based on the number of permutations [betapart_permutations] and the number of samples to include per permutation [betapart_samples]

- Note: permutation results are saved in *betapart/[mysample]–[distances]–[category]/permuations*

- *plots/Breakdown–[mysample]–[distances]–[category].svg*
  - Plot of dissimilarity components of the permutational values

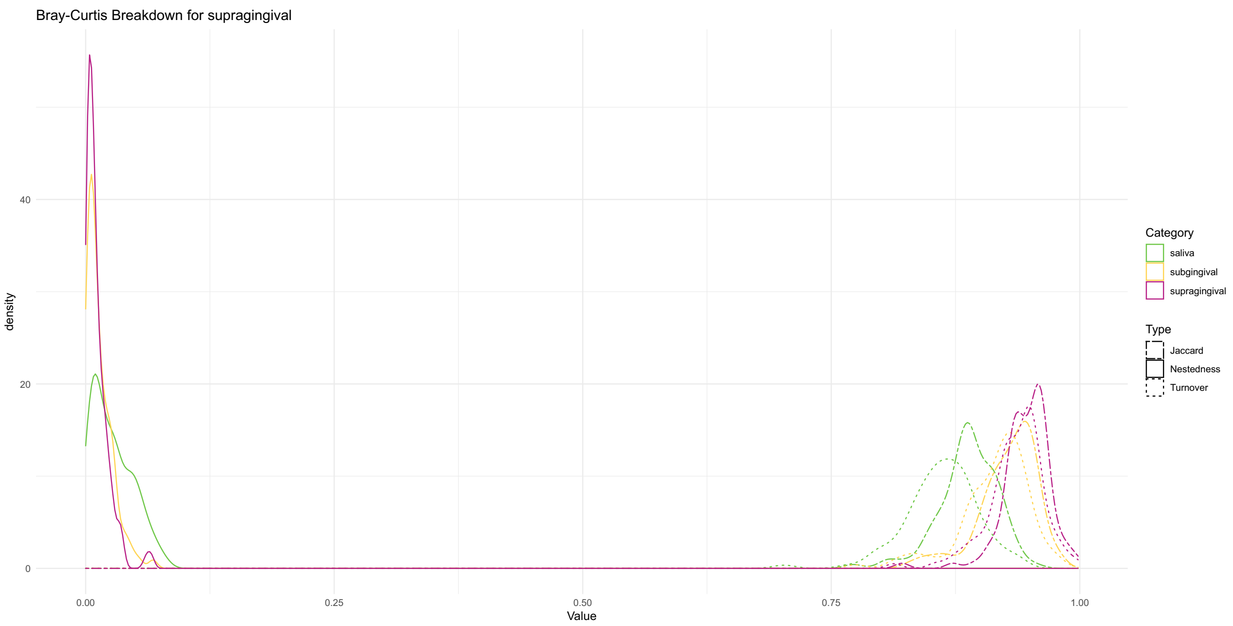

- *plots/betapart_pairwise/[mysample]–[distances]–[category]/[category:group1]+[category:group2].svg*
  - An alternate plot for pairwise group comparisons, based on the raw values of the two components.
    - Note: a .tsv file is saved in the same folder for the raw values

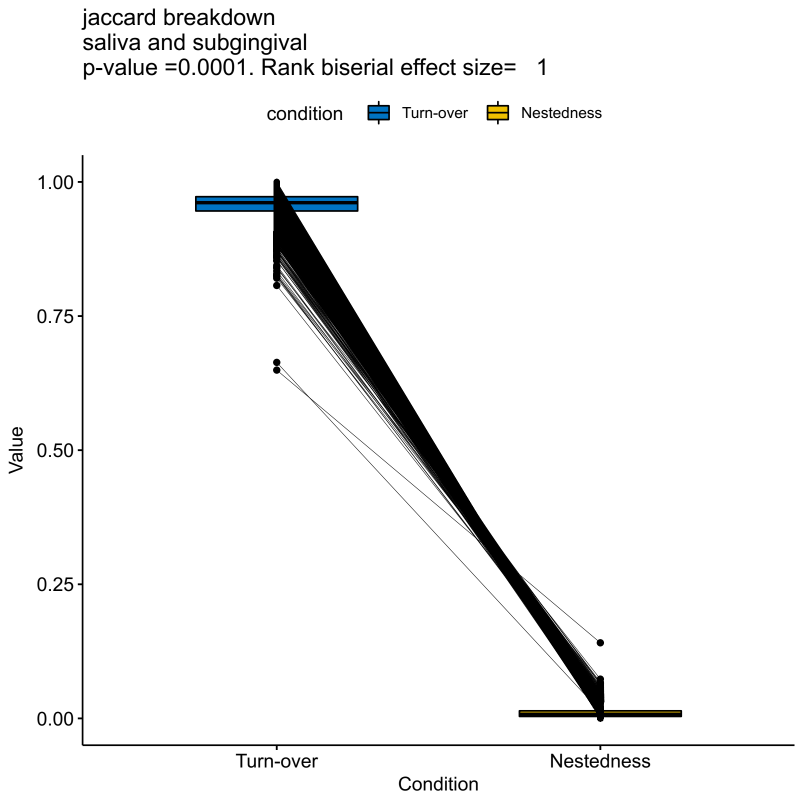
jaccard breakdown
saliva and subgingival
p-value =0.0001. Rank biserial effect size= 1
condition
Turn-over
Nestedness
1.00
0.75
0.50
0.25
0.00
Value
Turn-over
Nestedness
Condition

`network:` this command generates the following files:

This command generates network plots based on SparCC. The following will explain the steps done to generate the results, and what parameters in the falaphyl.yaml file are used.

Step 1: Core filtering

Filtering biom files to features available in each group in [category] to only those features that are present in [threshold] or more %. Core files are saved in:

- *network/[mysample]–[category]/core/[category:group1].tsv*

Step 2: SparCC calculations

Calculations are done for all pairwise samples. Files are saved in:

- *network/[mysample]–[category]/corr/[category:group1].tsv*

P-value calculations are done based on the specified number of permutations [sparcc_bootstrap]. The calculations are saved in:

- *network/[mysample]–[category]–corr/pvalue–[category:group1].tsv*

Step 3: Filtering and ZiPi value calculations

Filtering is done based on the desired level of relationship strength [sparcc_corr], and p-value [sparcc_pvalue].

Next, modularity is calculated using Louvain clustering from the R package igraph. These are used to calculate the importance of the nodes through its within module connectivity (Zi) and among modules connectivity (Pi). The saved files are formatted to be readily imported into Gephi as they are saved as nodes and edges files in the below locations.

- *network/[mysample]–[category]/nodes–[category:group1].tsv*
- *network/[mysample]–[category]/edges–[category:group1].tsv*

Step 4: ZiPi plotting

ZiPi values are plotted. Files are found in the following locations:

- *plots/network/ZiPi_[mysample]–[category]/ZiPi–[category:group1]*

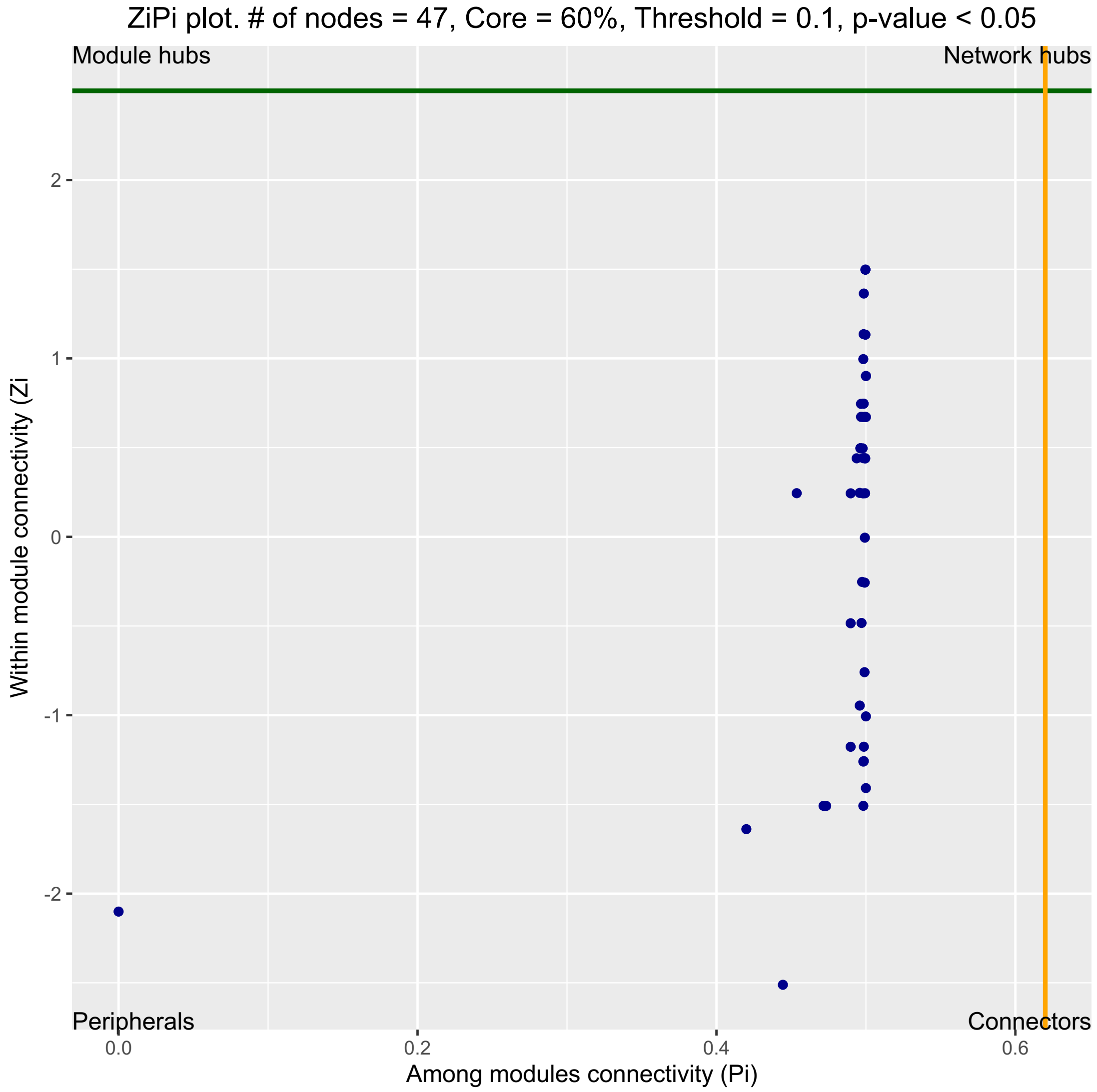

`diff`

This command generates differential abundances between groups in [category]. The command heavily uses the R package DAtest with some minor in-house changes that are explained below. To explore all the different differential abundance features that are available in this pipeline, refer to the falaphyl.yaml file under the [DA_tests] section. DA calculation is performed as follows:

Step 1: Feature prefiltering

Features are prefiltered prior to differential abundance calculations. Features that do not meet the following criteria are grouped together as a feature named “Other”. This is done to prevent loss of data that may skew the results due to the compositionality of the microbiome data.

- [DA_minsample]: The minimal number of samples a feature must be present for it to be retained.
- [DA_minread]: The minimal number of reads a feature must be present for it to be retained.
- [DA_minabund]: The fraction of the minimal mean relative abundance a feature must occupy for it to be retained.

Step 2: Differential abundance calculations

Each one of the tests in [DA_tests] is calculated separately after filtering. If [category] contains 3+ groups, then calculations are automatically generated for each pairwise groups.

In addition to the methods listed in [DA_tests] that are natively supported within the package, FALAPhyl also supports Compound Poisson Linear Models (CPLM) through its implementation in Tweedieverse. Calculations are saved in the below location:

- *diff/[mysamples]–[category]–minAbd[DA_minabund]minR[DA_minread]minS[DA_minsample]/diff–[category:group1]![category:group2]–[da_tests:test1].tsv*

Step 3: Area Under the Curve, False-Discovery rate, and Power calculations

DAtest package automatically calculates the suitability of each test for the dataset through its resilience in detecting the differential abundances if the data were spiked. FALAPhyl parallelizes the step to generate each spike, defined under [DA_effectsize]. Each spike is repeated 20 times.

Spike-in test results are saved in the following files:

- *diff/[mysamples]–[category]–minAbd[DA_minabund]minR[DA_minread]minS[DA_minsample]/AUC_FDR_Power/effectSize–[DA_effectSize]–[DA-tests].tsv*

Step 4: Plotting the graphs

The original plots of DAtest have been reimplemented. The following are the generated graphs with what was changed from the original DAtest implementation:

– plots/Score–[mysamples]–[category]–

minAbd[DA_minabund]minR[DA_minread]minS[DA_minsample].svg

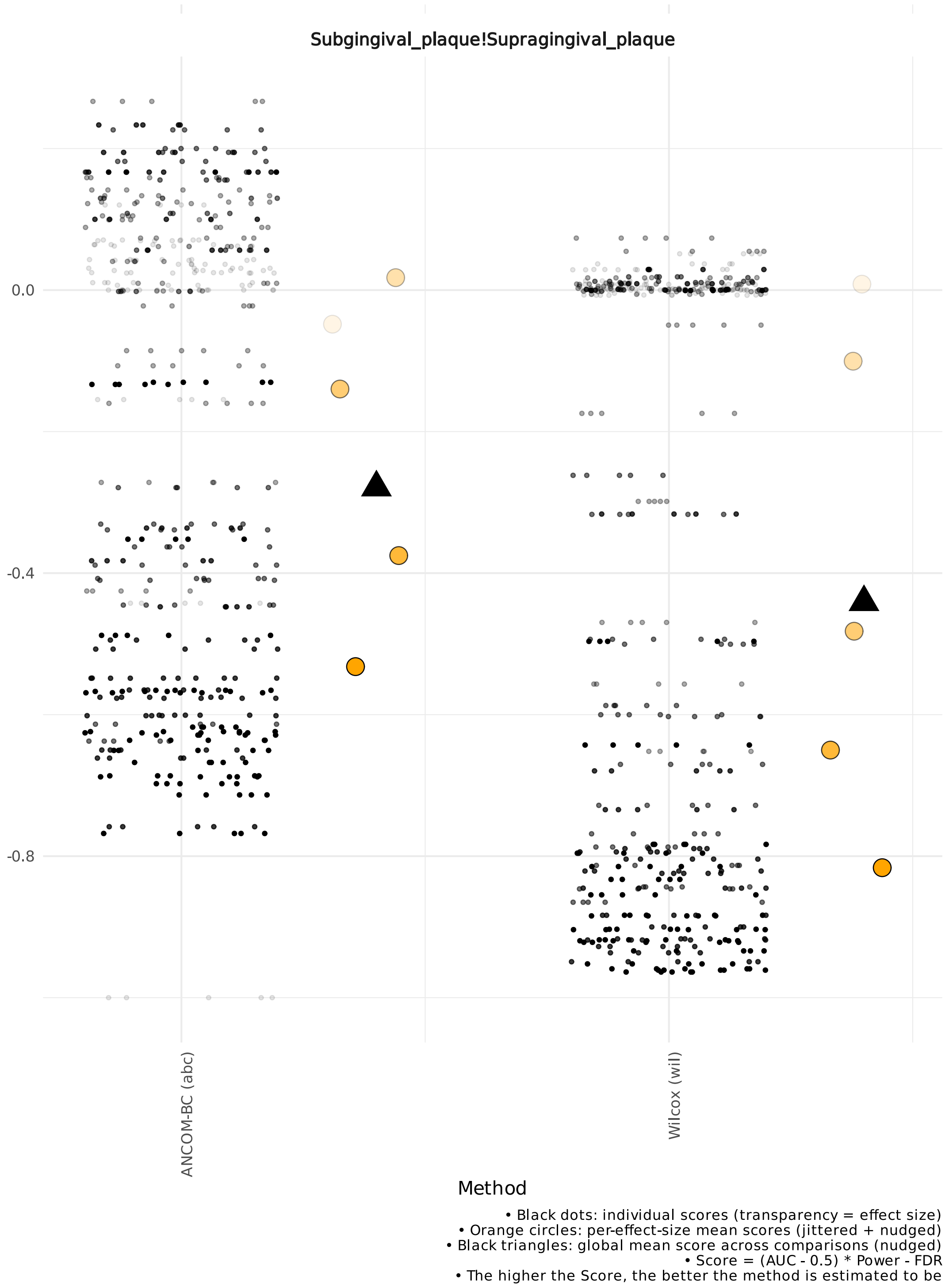

The averages of each spike used for the test’s effect size strength is added in the plot. Moreover, average of all spike tests is also added. Explanations of how scores are calculated are added to the bottom of the plot.

– plots/Power–[mysamples]–[category]–minAbd[DA_minabund]minR[DA_minread]minS[DA_minsample].svg

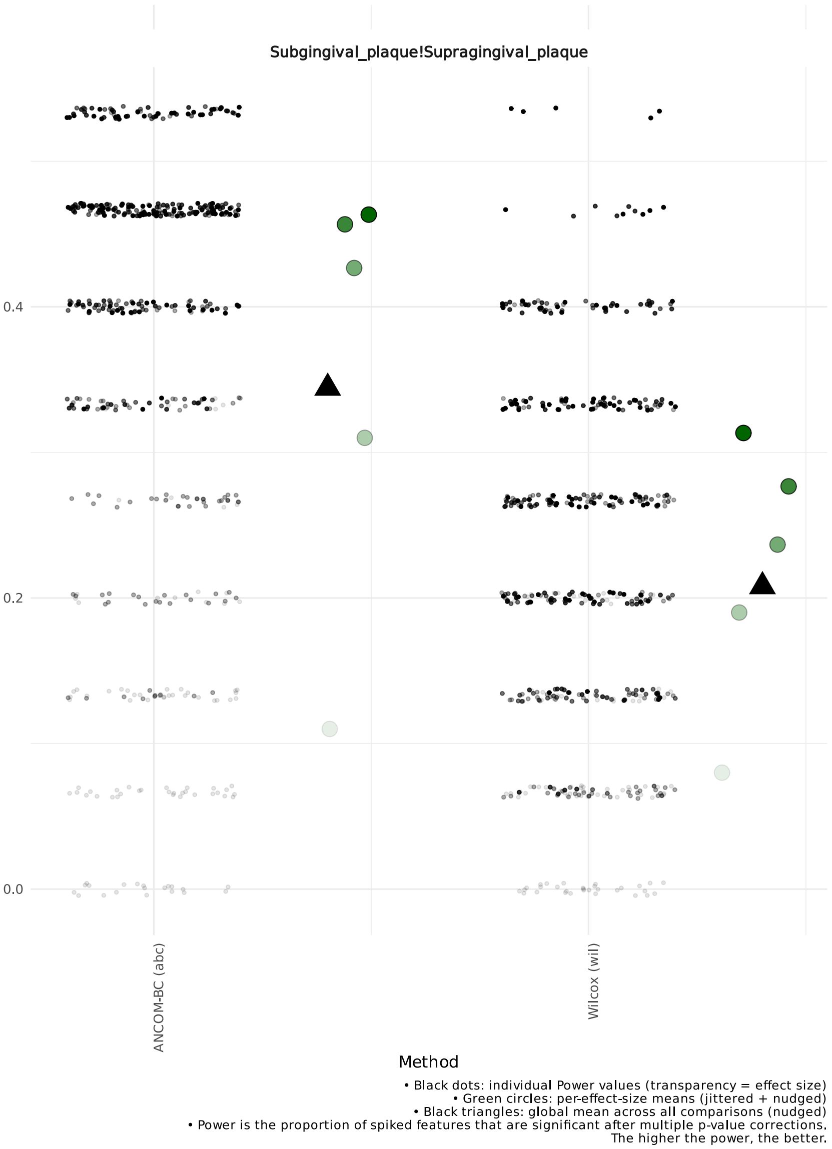

The averages of each spike used for the test’s effect size strength is added in the plot. Moreover, average of all spike tests is also added. Explanations of what power means in terms of a spike test is added.

– plots/FDR–[mysamples]–[category]–minAbd[DA_minabund]minR[DA_minread]minS[DA_minsample].svg

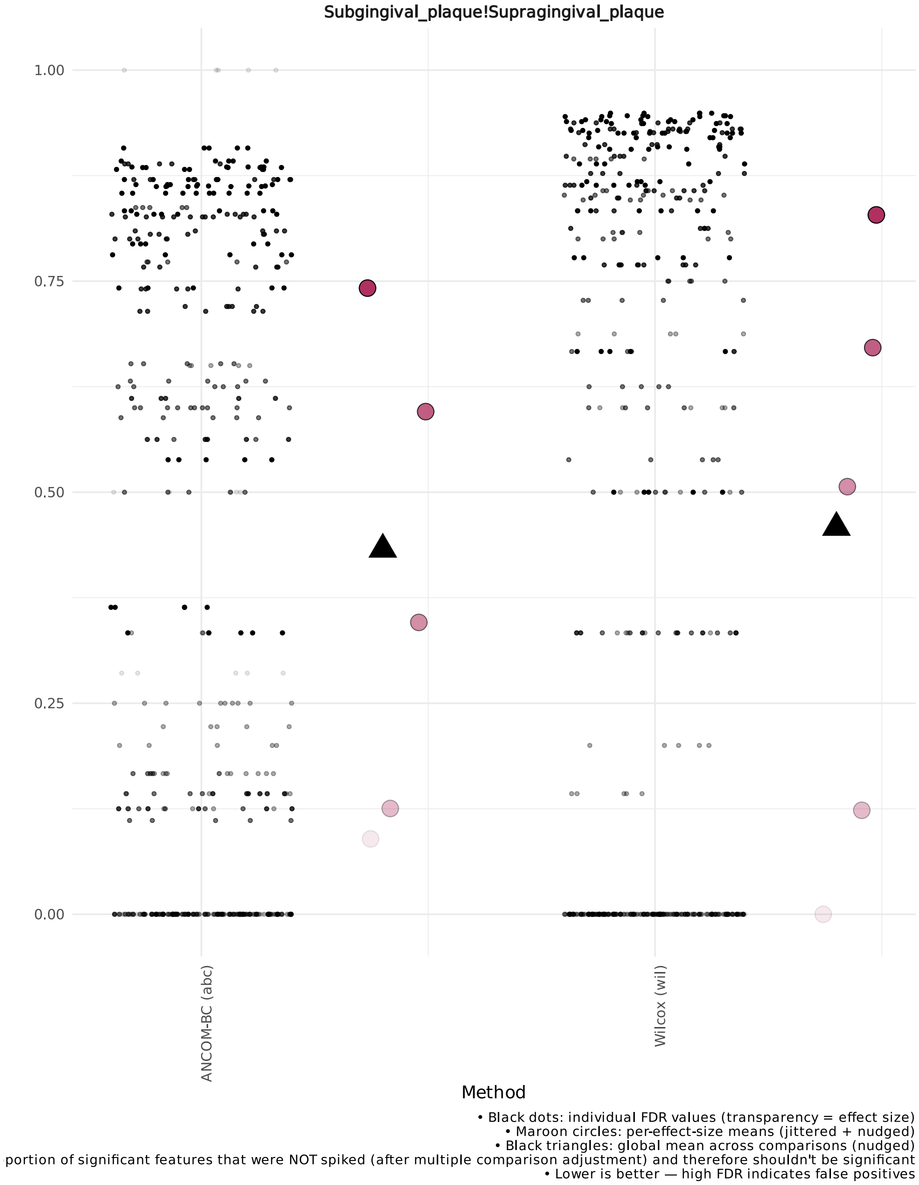

The averages of each spike used for the test’s effect size strength is added in the plot. Moreover, average of all spike tests is also added. Explanations of what FDR means in terms of a spike test is added.

– plots/AUC–[mysamples]–[category]–minAbd[DA_minabund]minR[DA_minread]minS[DA_minsample].svg

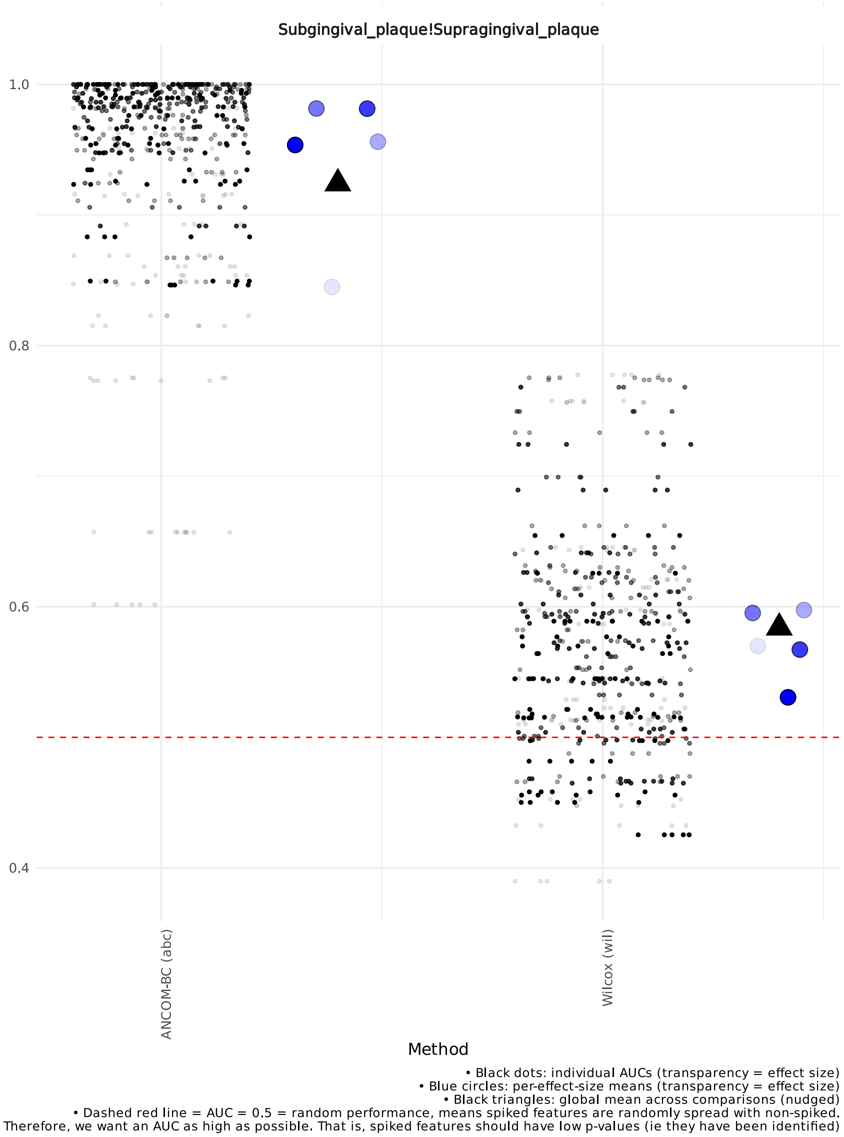

```
paired_diff
```

This command generates the same results as the above command, with the difference is that the groups are done at a sample-level, instead of group level. This is done by filtering [category] samples to only those that are present in [subjectID]. The files are saved in the same paths as above, with the only difference in the paths having the word “paired” in them.

`paired_alpha:` this command generates the following files:

- *plots/patientlvl_alphaDiv_[mysample]–[category]–[alpha]/[category:group1]_[category:group2].svg*

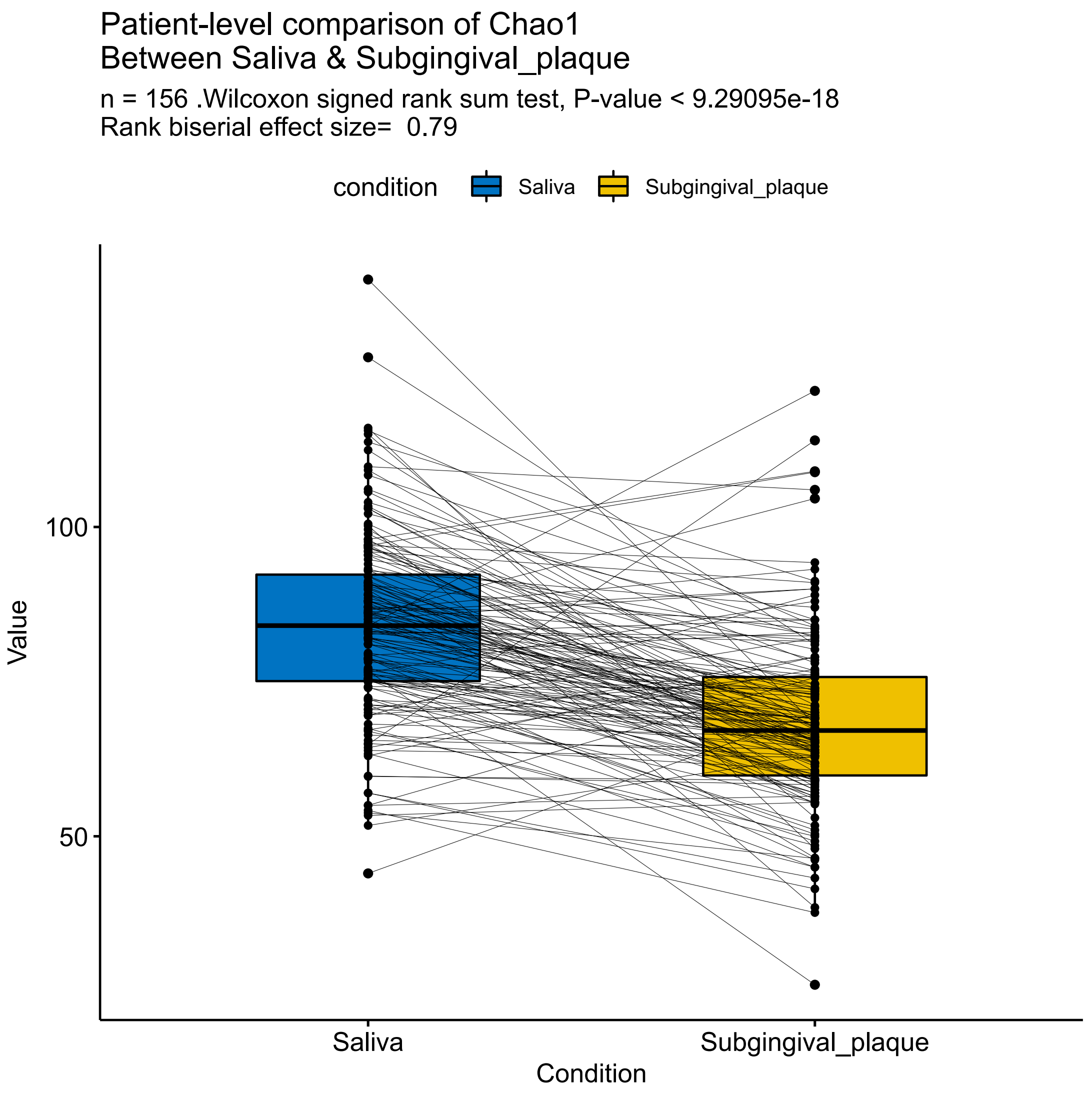

`paired_beta`

This command does the same analysis as `breakdown` per group, but restricted to only within the same [subjectID].

- *plots/patientlvl_alphaDiv_[mysample]–[category]–[distances]/[category:group1]_[category:group2].svg*

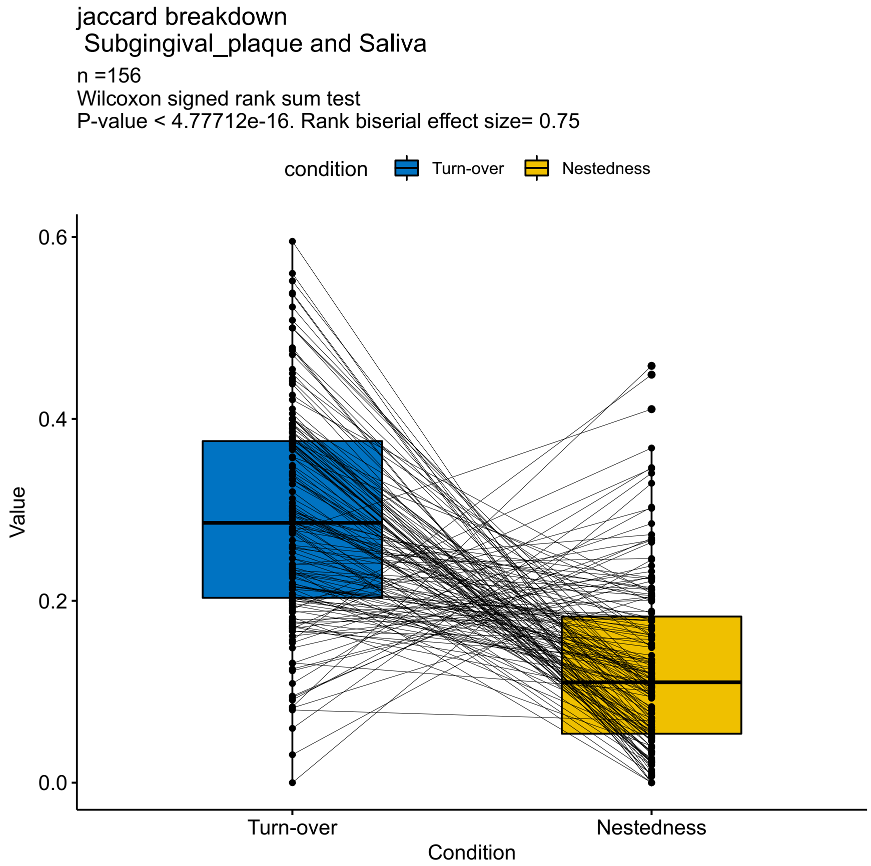

Appendix 2

Case study 2 materials and methods

Study Design and Participants The study was approved by the University of Alberta Research Ethics Board (Project No. Pro00120887). This 1-week interventional cohort pilot study recruited 14 families, each containing at least one preschool-aged child (five years old or younger) and at least one sibling. We chose this age for the youngest child to reduce outside-world contact associated with attending schools. Families were recruited from local community organizations such as churches and parent groups.

Sample Collection During the initial visit to the Oral Health Clinic at the University of Alberta, samples were collected from various oral sites of all family members. Participants were advised not to eat/drink for at least 30 minutes prior to the appointment. The following procedures were used for sample collection:

• Saliva: Unstimulated saliva samples were collected from adults using sterile tubes. The Micro•SAL™ device (Oasis Diagnostics, Vancouver, Washington, USA) was used for children. The device was placed in the mouth for a set period to absorb saliva when the sampler was saturated, after which saliva was extruded from the cotton and stored at -20°C.

• Buccal Mucosa and tongue: The inside of the cheek (buccal mucosa) was sampled using the DNA•SAL™ device (Oasis Diagnostics). Participants were asked to scrub the buccal mucosa and store it in the solution provided with the device at -20C. Cells were removed from the device by vortexing in 1X PBS and lysozyme for 1 hour. The supernatant was then used for downstream DNA isolation. Tongue samples were collected similarly.

• Supragingival Plaque: Supragingival plaque was collected using sterile paper points, which were gently placed at multiple supragingival sites and held in place for 20 seconds. All collected points were pooled in a single tube containing RNAlater for storage at -20°C.

• Subgingival Plaque: Subgingival plaque was collected from the distal surfaces of the lower central incisors using sterile paper points. The points were inserted subgingivally into the sulcus for 20 seconds, then transferred to a tube containing RNAlater for preservation and storage at -20°C. Following the baseline sample collection, each family's test subject underwent professional oral prophylaxis to disrupt the existing biofilm in the oral cavity. This procedure involved a comprehensive cleaning of the teeth and gums using rubber cups and pumice to disrupt and remove the existing biofilm, effectively displacing the oral bacteriome. A questionnaire (supplementary) was given to the parents to document familial demographic characteristics, and close contact activities among the family members. Subgingival samples were collected from the test participant one week after the prophylaxis using the same procedure described above.

DNA Extraction and Sequencing DNA was extracted from the collected samples using the QIAGEN QIAamp DNA Mini Kit (QIAGEN, Germantown, MD, USA), following the manufacturer's protocol. The concentration and purity of the extracted DNA were assessed using a Qubit 4 Fluorometer (ThermoFisher Scientific, Waltham, MA, USA) with the Qubit™ 1x dsDNA High-Sensitivity Assay Kit, ensuring that the DNA was of sufficient quality for subsequent sequencing. The V1-V3 regions of the 16S rRNA gene were amplified using specific primers (27F: 5'-ACACTCTTTCCCTACACGACGCTCTTCCGATCTGAAKRGTTYGATYNTGGCTCAG-3'and 519R: 5'-GTGACTGGAGTTCAGACGTGTGCTCTTCCGATCTACGTNTBACCGCDGCTGCTG-3'). The amplified

DNA was then sequenced at the Genome Quebec core facility. FASTQ files submitted to NIH SRA (PRJNA1159177).

Appendix 3

Case study 3 materials and methods

Participants and Study Design

Participants were recruited from the general community and from the student body of the Faculty of Dentistry at Dentistry Dalhousie University in Halifax, Nova Scotia, Canada to participate in a pilot, parallel arm randomized clinical trial. Study protocol review and ethical approval were obtained from the Dalhousie University Research Ethics Board (REB# 2024-7166). The study protocol and plan for analyses were registered as a clinical trial at ClinicalTrials.gov (identifier: NCT06588049) prior to recruitment and study commencement. Sequences are deposited in NIH SRA (project ID PRJNA1300299).

Recruitment efforts included email invitations and printed posters circulated within the Faculty. Eligible participants were healthy individuals classified as ASA I or II, aged 18 to 40 years on the day of follow-up. Exclusion criteria included pregnancy, uncontrolled systemic diseases or acute infections, use of probiotics, systemic antibiotic or antimicrobial mouthwash use within the past month. Participants with documented allergy to soy, nuts, seeds, dairy, egg products, fish, shellfish or wheat were excluded. The presence of acute or chronic oral conditions including herpes simplex virus, stomatitis, oral lichen planus, angular cheilitis, candidiasis, necrotizing gingival diseases, active systemic infection, active dental caries, or any periodontal diagnosis other than “health periodontium”, or “localized gingivitis” as defined by the European Federation of Periodontology, 2019.

A sample size of 30 participants (15 per group) was initially targeted to detect a statistically significant difference with a hypothesized effect size of 0.4, using a two-tailed test with 5% significance and 80% power, calculated using OpenEpi® Version 3.01. Following screening, 21 eligible participants were enrolled in the parallel arm randomized clinical trial and assigned to either the intervention group (n = 11; nitrate mouthrinse) or the placebo group (n = 10) using a computer-generated random sequence. The allocation sequence was created using a simple randomization method in Microsoft Excel. To ensure allocation concealment, the randomization sequence was generated by an independent researched who was not involved in participant recruitment or data collection.

All study visits, including baseline and follow- up assessments, were conducted at the Faculty of Dentistry clinic. The trial commenced on August 14, 2024, and concluded on November 27, 2024.

Participants in the intervention group were instructed to swish 15mL of a nitrate-rich mouthrinse containing 7.5g of Bio-Steel ® Sport Beets (NPN:80104749) in 250mL of sterile, distilled water, and then expectorate after 30 seconds, once daily for 14 consecutive days, following their usual nightly oral hygiene routine. Participants in the control group were instructed to swish with 15mL sterile, distilled water for 30 seconds, and then expectorate. Participants were instructed to maintain their typical diets and oral care practices throughout the study period.

Data Collection and Analysis

At the baseline visit, participants were screened for eligibility criteria and underwent an oral examination to screen for oral pathologies, caries, and periodontal disease.

The primary outcome variable of oral microbiome was collected as tongue swab samples using the OMNIGENE® ORAL (OMR-120) collection kit. A secondary outcome of salivary nitric oxide concentrations was evaluated using HumanN® nitric oxide indicator strips according to the manufacturer's protocol. The resulting colorimetric readings were compared against a reference legend and categorized on a 3-point scale: depleted, low, or optimal. Standardized photographs of the strips alongside the reference legend were reviewed by two blinded examiners. In cases of disagreement, a third blinded examiner provided the final assessment. Blood pressure was collected as an exploratory variable using a calibrated manual sphygmomanometer and a standard stethoscope, with participants seated and rested for at least 5 minutes prior to measurement. Three readings were taken at 5-minute intervals, with the average value used for analysis in accordance with American Heart Association guidelines.

Follow-up visits occurred exactly 14 days after the baseline visit and at approximately the same time of day to control for diurnal variation.

Tongue swab samples were submitted for 16S rRNA sequencing to characterize and compare the microbial community structure between groups. DNA extraction and quantification were conducted by Genome Québec. A total of 42 bacterial libraries (16S V3–V5, using primers 515bF–926R) and 42 libraries (16S V1–V3, using primers 27Fb–519R), along with one PCR blank per set,

were prepared. A standard bioinformatics pipeline was employed, including analyses of alpha and beta diversity, and differential relative abundance of taxa between the intervention and placebo groups.
